# Evaluation of Technology-Enabled Monitoring of Patient-Reported Outcomes to Detect and Treat Toxic Effects Linked to Immune Checkpoint Inhibitors

**DOI:** 10.1001/jamanetworkopen.2021.22998

**Published:** 2021-08-30

**Authors:** Pavlos Msaouel, Clara Oromendia, Arlene O. Siefker-Radtke, Nizar M. Tannir, Sumit K. Subudhi, Jianjun Gao, Yinghong Wang, Bilal A. Siddiqui, Amishi Y. Shah, Ana M. Aparicio, Matthew T. Campbell, Amado J. Zurita, Leah K. Shaw, Lidia P. Lopez, Heather McCord, Sandip N. Chakraborty, Jacqueline Perales, Cong Lu, Michael L. Van Alstine, Michael Elashoff, Christopher Logothetis

**Affiliations:** 1 Division of Cancer Medicine, Department of Genitourinary Medical Oncology, The University of Texas MD Anderson Cancer Center, Houston; 2 Division of Pathology and Laboratory Medicine, Department of Translational Molecular Pathology, The University of Texas MD Anderson Cancer Center, Houston; 3 The Ronin Project, San Mateo, California; 4 Division of Internal Medicine, Department of Gastroenterology, Hepatology, and Nutrition, The University of Texas MD Anderson Cancer Center, Houston; 5 Division of Cancer Medicine, The University of Texas MD Anderson Cancer Center, Houston

## Abstract

**Importance:**

Immune checkpoint inhibitors can produce distinct toxic effects that require prompt recognition and timely management.

**Objective:**

To develop a technology-enabled, dynamically adaptive protocol that can provide the accurate information needed to inform specific remedies for immune toxic effects in patients treated with immune checkpoint inhibitors.

**Design, Setting, and Participants:**

An open-label cohort study was conducted at a single tertiary referral center from September 6, 2019, to September 3, 2020. The median follow-up duration was 63 (interquartile range, 35.5-122) days. Fifty patients with genitourinary cancers treated with immune checkpoint inhibitors were enrolled.

**Interventions:**

A fit-for-purpose electronic platform was developed to enable active patient and care team participation. A smartphone application downloaded onto patients’ personal mobile devices prompted them to report their symptoms at least 3 times per week. The set of symptoms and associated queries were paired with alert thresholds for symptoms requiring clinical action.

**Main Outcomes and Measures:**

The primary end point of this interim analysis was feasibility, as measured by patient and care team adherence, and lack of increase in care team staffing. Operating characteristics were estimated for each symptom alert and used to dynamically adapt the alert thresholds to ensure sensitivity while reducing unnecessary alerts.

**Results:**

Of the 50 patients enrolled, 47 had at least 1 follow-up visit and were included in the analysis. Median age was 65 years (range, 37-86), 39 patients (83%) were men, and 39 patients (83%) had metastatic cancer, with the most common being urothelial cell carcinoma and renal cell carcinoma (22 [47%] patients each). After initial onboarding, no further care team training or additional care team staffing was required. Patients had a median study adherence rate of 74% (interquartile range, 60%-86%) and 73% of automated alerts were reviewed within 3 days by the clinic team. Symptoms with the highest positive predictive value for adverse events requiring acute intervention included dizziness (21%), nausea/vomiting (26%), and shortness of breath (14%). The symptoms most likely to result in unnecessary alerts were arthralgia and myalgia, fatigue, and cough.

**Conclusions and Relevance:**

The findings of this cohort study suggest an acceptable and fiscally sound method can be developed to create a dynamic learning system to detect and manage immune-related toxic effects.

## Key Points

**Question:**

Can a technology-enabled, dynamically adaptive protocol be efficiently used to provide early and accurate detection of toxic effects to immune checkpoint inhibitors?

**Findings:**

In this cohort study including 47 individuals with genitourinary cancers, a median patient adherence rate of 74% and a care team–automated alert review rate of 73% within 3 days without incurring the cost of increasing care team staffing was found. Dizziness, nausea and vomiting, and shortness of breath were the symptoms with the highest positive predictive value for adverse events requiring acute interventions.

**Meaning:**

The findings of this study suggest that technology-enabled monitoring of patient-reported outcomes may provide a useful model for delivering complex care remotely in patients receiving immune checkpoint inhibitors.

## Teaser

This cohort study conducts an interim analysis of an electronic platform developed for patients to report symptoms possibly related to the toxic effects of immune checkpoint inhibitors.

## Introduction

Immune checkpoint inhibitors (ICIs) can produce a wide range of distinct immune-related toxic effects that pose unique challenges, because they can occur late after treatment initiation, involve multiple organs, and require specific remedies.^1,2,3^ The successful management of these toxic effects requires prompt collaboration between medical specialties. Early and accurate recognition of toxic effects leads to timely and specific interventions that are necessary to reduce the risk of potentially life-threatening complications and minimize interruption of ICI therapy.^3^ Toward this goal, technology-enabled remote monitoring of immune toxic effects can result in generalizable improvements in outcomes that will accelerate the safe application of ICIs by the general oncology community.

Studies performed before the broad adoption of ICIs in oncology have reported that electronic symptom monitoring using patient-reported outcomes (PROs) during routine cancer care can produce clinical benefit, including increased overall survival.^4,5,6,7,8,9,10,11^ However, a recent pilot study found that the use of an electronic PRO tool did not reduce the number of adverse events in patients with ICI.^12^ The platform used was not available on mobile devices, did not directly alert the care teams, and did not use adaptive alert thresholds to maximize sensitivity and minimize excess alerts. We hypothesized that an electronic interface accessible via mobile devices might facilitate prompt and useful bidirectional communication between patients at risk for immune toxic effects and their health care teams. To achieve this goal, several considerations must be accounted for, including data security, patient adherence, adaptability for use in living environments, and reliable and efficient communication of PROs to the care team.^13^ To address these considerations, we initiated a technology-enabled, dynamically adaptive protocol designed to provide the accurate information needed to inform specific remedies for immune toxic effects in patients treated with ICIs. We conducted the present preplanned analysis of the first 50 patients enrolled to evaluate the feasibility of our electronically enabled infrastructure.

## Methods

### Study Design and Participants

This open-label protocol enrolled patients with genitourinary cancers treated with ICIs at The University of Texas MD Anderson Cancer Center. The present interim analysis was conducted in patients recruited from September 6, 2019, to September 3, 2020. The study was approved by The University of Texas MD Anderson Cancer Center Institutional Review Board. All participants provided written informed consent; they did not receive financial compensation. This protocol has been developed according to the Standard Protocol Items: Recommendations for Interventional Trials (SPIRIT) reporting guideline 2013.^14^ The present report followed the Strengthening the Reporting of Observational Studies in Epidemiology (STROBE) reporting guideline for cohort studies. Additional details are provided in the eMethods in the Supplement regarding the electronic infrastructure, patient eligibility, data collection, encryption/security standards, consensus events meriting alerts, dynamic alert thresholds, and the methods applied to identify the set of electronic questions and their initial alert thresholds.

At baseline (within 1 week before ICI initiation) and between ICI treatment visits, patients were prompted on their smartphones via a notification by the smartphone app to complete the symptom check-in a minimum of 3 times per week. Examples of push notifications are shown in eAppendix 1 in the Supplement. Patients were able to report additional symptoms outside of the scheduled prompt times and symptom types in the event that they wanted to log symptoms between visits. Patients not reporting 3 times per week received phone call reminders from research staff, who also asked the patients about potential barriers to reporting.

Each time a particular symptom report triggered a prespecified symptom alert threshold (eAppendix 2 in the Supplement), the remote monitoring app informed patients to call their clinical care team and simultaneously sent automated emails to the clinical care team. Patients who developed symptoms likely attributable to ICI toxicity were evaluated and managed using a standardized approach shown in eAppendix 3 in the Supplement that was developed based on standard guidelines and expert consensus.^15^ The end of ICI therapy was defined as the date of the first follow-up appointment for patients in therapeutic clinical trials or 28 days after the last immunotherapy treatment for patients receiving standard care. Patients were followed up for 100 days after the end of ICI therapy. During this follow-up period, patients could continue reporting symptoms but were no longer required to report 3 times per week. After the end of the follow-up period, patients were removed from the study and were no longer able to report symptoms via the smartphone app.

### Outcome Definitions

The primary end point of this interim analysis was feasibility as determined by 3 metrics focusing on patient and care team acceptability and demand, as well as organizational implementation^16^: (1) patient adherence, (2) care team adherence, and (3) no increase in care team staffing. A patient was deemed adherent on a particular day if they had completed a survey on that day or within the previous 2 days. Adherence was summarized by the mean of the percent of days adherent for each patient. The overall percent of days adherent was reported. Because some patients had longer follow-up times than others, mean patient adherence was also studied. Care team adherence was measured by the time to respond to an alert. The percent of responses within 3 days and 7 days was reported. From an organizational implementation standpoint, the protocol was expected to be used as designed and without requiring any increase in care team staffing. No hypothesis testing was prespecified to compare the feasibility metrics with specific thresholds.

### Statistical Analysis

Demographic and clinical characteristics of patients were summarized as appropriate. Comorbidities were identified from diagnostic codes using the Elixhauser method, a well-validated method of classifying and weighting comorbid conditions based on *International Statistical Classification of Diseases, Version 10*, diagnosis codes.^17^ The Elixhauser comorbidity index level was estimated based on the active problems the patients had at the time of study enrollment. The primary end point of the final analysis will be association between the symptom alerts and mitigation of ICI toxic effects, and the trial was powered to perform this analysis when 100 patients have been accrued. The present interim feasibility analysis was prespecified to be performed at the accrual midpoint, once patients were enrolled, with no formal hypothesis-based power analysis of feasibility metrics. The formulas used for positive predictive value, negative predictive value, sensitivity, and specificity are listed in eAppendix 5 in the Supplement. Statistical analysis was performed with R, version 3.6.3 (R Foundation)

## Results

### Individual Patient Care

The study was initiated on September 6, 2019. At the time of data lock on September 3, 2020, 50 patients were enrolled and 47 had at least 1 follow-up visit and were included in this analysis (eFigure 1 in the Supplement). All eligible patients who were approached to enroll agreed to participate. Clinical and demographic details can be found in Table 1. The median age was 65 years (range, 37-86). Eight patients (17%) were women, 39 patients (83%) were men, and 39 patients (83%) had metastatic cancer. The most common primary cancers were urothelial cell carcinoma (22 [47%]) and renal cell carcinoma (22 [47%]), although prostate (1 [2%]) and urethral (2 [4%]) carcinomas were also present. Enrolled patients had the expected moderate level of comorbidities for their age group, with 50% having an Elixhauser comorbidity index score between 3 and 6. Individual components can be found in eTable 1 in the Supplement. The baseline smart phone use characteristics of enrolled patients are reported in eTable 2 in the Supplement.

**Table 1.** Demographic, Clinical, and Treatment Characteristics of 47 Patients Included in Analysis

Characteristic	No. (%)
Sex	
Female	8 (17)
Male	39 (83)
Race	
Black or African American	2 (4.3)
White	41 (87)
Other^a^	4 (8.5)
Age, median (IQR), y	65 (61, 71)
Range	37-86
Elixhauser comorbidity index score, median (IQR)	5.00 (3.00-6.00)
Range	2.00-11.00
Primary cancer	
Prostate carcinoma	1 (2.1)
Renal cell carcinoma	22 (47)
Urethral carcinoma	2 (4.3)
Urothelial carcinoma	22 (47)
Drugs included in the ICI regimen	
PD1 inhibitor	41 (91)
PD-L1 inhibitor	1 (2.2)
CTLA-4 inhibitor	11 (24)
Interleukin-2 pathway therapy	14 (31)
Tyrosine kinase inhibitor	13 (29)
Cytotoxic chemotherapy	2 (4.4)
Combination of the above	37 (82)

Abbreviations: ICI, immune checkpoint inhibitor; IQR, interquartile range.

^a^ No further breakdown available.

All enrolled patients underwent treatment with ICIs. Combination of an ICI with another immunotherapy or other agents, such as tyrosine kinase inhibitors was given in 37 patients (79%). The ICI regimen included an anti-PD1 drug in 41 patients (87%), a drug targeting the interleukin-2 pathway in 14 patients (30%), and an anti–CTLA-4 drug in 11 patients (23%). Thirteen patients (28%) received tyrosine kinase inhibitors in combination with ICIs (Table 1).

At the time of data lock, 18 (38%) patients had completed treatment, and 29 (62%) were still receiving an ICI. Patients were followed up for a median of 63 (interquartile range [IQR], 35.5-122) days. A total of 1502 surveys were completed by the 47 patients over 4552 patient-days. Patients submitted a median of 24 surveys (IQR, 2-9) and had a median study adherence rate of 74% (IQR, 60%-86%). Patient adherence was generally consistent throughout the study period. All patients submitted surveys at least once per month while receiving ICI therapy. Patient adherence rates stratified by sex and age group are shown in eFigure 2 in the Supplement.

Symptom reports led to 409 automatic alerts. The median response time from the care team was 19 hours (IQR, 3-80 hours), and 297 (73%) of these alerts were reviewed within 3 days (89% reviewed within 7 days). Because an alertable report could contain 1 or more individual alertable symptoms, these 409 symptom reports corresponded to 593 individual symptom alerts. Throughout follow-up, 139 144 recorded telephone encounters pertained to symptom management (mean [SD], 1.39 [2.13] calls per patient-month). Of these calls, 18 (13%) required physician involvement, and the rest included only other members of the care team.

The most frequently reported symptoms in the 47 patients were fatigue (40%), pain (21%), and diarrhea (21%). Arthralgia and myalgia, although less frequently reported (17% of all surveys), were the most frequently alerted symptoms, producing 142 alerts. All symptoms and their frequency are listed in Table 2.

**Table 2.** Frequency of Symptom Reporting and Alerts Triggered

Symptom	Alerts, No. (% of reports completed)^a^	Symptom reported, No. (% of reports completed)^a^
Arthralgia and myalgia	142 (9.45)	248 (16.51)
Pain	116 (7.72)	322 (21.44)
Fatigue	80 (5.33)	600 (39.95)
Shortness of breath	64 (4.26)	114 (7.59)
Cough	48 (3.20)	121 (8.06)
Abdominal pain	39 (2.60)	134 (8.92)
Diarrhea	32 (2.13)	318 (21.17)
Fever	27 (1.80)	53 (3.53)
Dizziness	19 (1.26)	100 (6.66)
Nausea and vomiting	12 (0.80)	129 (8.59)
Dysuria	9 (0.60)	35 (2.33)
Concentration and memory issues	3 (0.20)	9 (0.60)
Palpitations	2 (0.13)	6 (0.40)
Anxiety	0	96 (6.39)
Blurry vision	0	68 (4.53)
Pruritus	0	294 (19.57)

^a^ Percentages based on 1502 surveys completed over 4552 patient-days.

### Observations Across Patients and Alert Updates

The detailed longitudinal nature of our data collection allowed for visualization of symptoms throughout treatment (Figure; and eFigures 3, 4, and 5 in the Supplement). As shown in eFigure 6 and eTable 3 in the Supplement, some symptoms tended to be present for longer (pain, blurry vision, rash/itchy skin), and others occurred in shorter episodes (cough, fever, racing heartbeat). Adverse events that occurred in patients during the study are shown in eFigure 7 in the Supplement.

**Figure.**
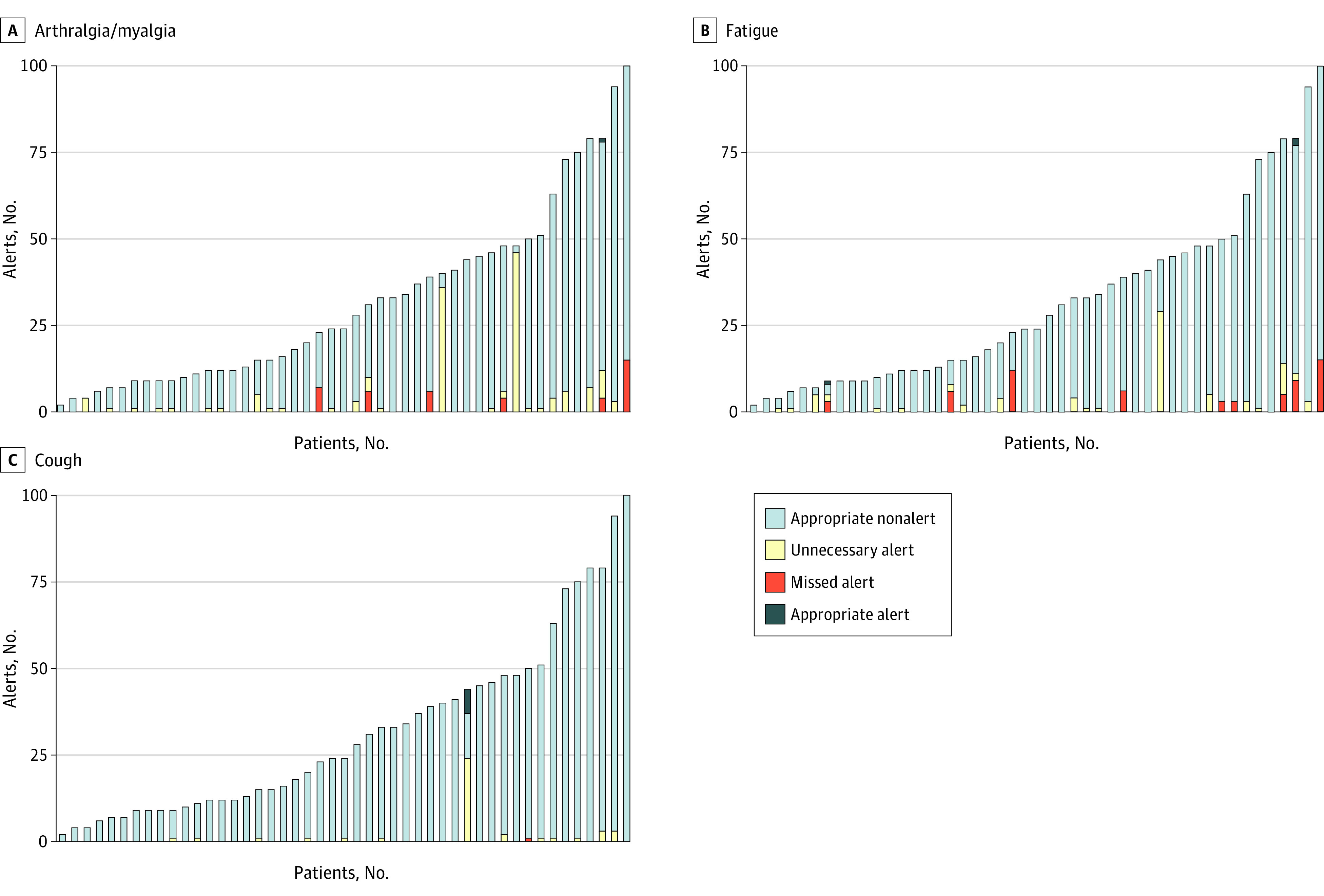
Total Number of Alerts per Patient Collected Over Time for the 3 Symptoms That Required Alert Threshold Updating Due to High Frequency of Unnecessary Alerts Relative to Missed Alerts The 3 symptoms were arthralgia and myalgia (A), fatigue (B), and cough (C). Appropriate alerts were linked to clinical interventions. Appropriate nonalerts were defined as the absence of prompts following symptom reports that were not linked to clinical interventions. Unnecessary alerts were alerts following symptom reports that were not linked to clinical interventions. Missed alerts were defined as the absence of prompts following symptom reports that were linked to clinical interventions.

Of the 593 symptom alerts, 51 (9%) were linked to an adverse event and clinical intervention and thus deemed to be an appropriate alert. The weighted overall positive predictive value was 6.9% (95% bootstrap CI, 5.8%-8.2%), with dizziness (21%), nausea and vomiting (26%), and shortness of breath (14%) having the highest positive predictive values. Operating characteristics for each symptom are listed in Table 3.

**Table 3.** Operating Characteristics Under Initial and Updated Symptom Alert Thresholds

Symptom	Initial Threshold			Updated Threshold			Changes				
	Alerts, No.	PPV, %	NPV, %	Alerts, No.	PPV, %	NPV, %	Appropriate alerts removed, No.	Unnecessary alerts removed, No.	Alerts removed, %	PPV improvement, %	NPV improvement, %
Arthralgia and myalgia	142	0.58	100	44	6.69	100	0	98	69	1.056	0
Pain	116	0.19	89.64	116	10.19	89.64	0	0	0	0	0
Fatigue	80	5.54	94.97	47	7.48	95.10	0	33	41	35	0
Shortness of breath	64	14.19	100	64	14.19	100	0	0	0	0	0
Cough	48	1.38	100	46	1.52	100	0	2	4	10	0
Abdominal pain	39	6.17	100	39	6.17	100	0	0	0	0	0
Diarrhea	32	9.89	95.88	32	9.89	95.88	0	0	0	0	0
Fever	27	5.67	100	27	5.67	100	0	0	0	0	0
Dizziness	19	21.03	93.94	19	21.03	93.94	0	0	0	0	0
Nausea and vomiting	12	26.17	89.17	12	26.17	89.17	0	0	0	0	0
Dysuria	9	0	86.84	9	0	86.84	0	0	0	0	0
Concentration and memory issues	3	0	100	3	0	100	0	0	0	0	0
Palpitations	2	0	100	2	0	100	0	0	0	0	0

Abbreviations: NPV, negative predictive value; PPV, positive predictive value.

At the time of analysis, 7 of 16 (44%) symptoms (with 15 total subquestions) had accrued sufficient data to allow for consideration of alert changes. Alerts were found to be unnecessary at the thresholds originally chosen for 7 questions (eTable 4 in the Supplement), accounting for 18% of all alerts triggered. These changes were in 3 symptoms: arthralgia and myalgia, fatigue, and cough; 69% (98 of 142) of alerts for arthralgia and myalgia could be removed without reducing sensitivity. Further refinement of alert thresholds will be pursued at the completion of follow-up.

### Communication Between Patients and Care Teams

Remote symptom reporting was incorporated into established care teams, enabling a new form of communication between patients and care teams without additional staffing (eFigure 8 in the Supplement). After initial training, no further care team training was required and alert resolution was not identified as a major time burden. Standardization of symptom descriptions from patients allowed automatic triaging of reports, which concentrated care team time on patients in need. Telephone encounter notes revealed that, although in some cases an immediate visit to the clinic was warranted, advice on simple steps to mitigate symptoms (eg, more fluid intake, rest), as well as resetting of expectations on symptoms, was often sufficient. Example telephone encounter notes can be found in eAppendix 4 in the Supplement.

## Discussion

Our results appear to support the hypothesis that technology-enabled integration of PROs via the platform we developed enables electronic monitoring followed by automated alerts and specific responses to toxic effects in patients treated with ICIs. Adherence to the protocol by patients and care teams suggests that this technology-enabled platform addressed an unmet need. Our study evaluated both the adherence rate with symptom reporting at least 3 times per week and the review rate of automated alerts by the care team within 3 days. These distinct feasibility requirements hinder direct historical comparisons with previously reported adherence rates. However, the 74% median patient-level adherence rate of symptom reporting at least 3 times per week and the 73% care team automated alert review rate are within the expected range of what has previously been observed with electronic PRO symptom reporting in oncology.^10,12,18,19,20,21^ For example, a feasibility trial found that participants with early-stage breast cancer who were receiving aromatase inhibitor therapy noted a weekly adherence rate of 38% with use of a web-based application, which increased to 74% with the addition of a weekly reminder.^19^ Our study enrollment overlapped with the COVID-19 pandemic, which can confound comparisons with historical adherence expectations but also suggests that the platform demonstrated reasonable adherence rates by both patients and care teams despite the operational challenges imposed by the pandemic. Fiscally sound engagement of the clinical environment is a necessary component for broad adoption of our initiative. With this in mind, we established the operational parameter that implementation of our protocol would not require increasing care team staffing. Consequently, any efficiencies gained can more confidently be attributed to the new infrastructure. Our platform was feasibly implemented in a traditionally organized clinical center without incurring the cost of adding new staff, thus supporting widespread application.

This technology-enabled platform was developed and initially implemented in the department of genitourinary medical oncology to address the challenges imposed by toxic effects induced by US Food and Drug Administration–approved ICIs in urothelial and renal cell carcinomas, as well as ongoing clinical trials of ICI-based combinations in all genitourinary cancers. High dose interleukin-2 is not administered in the department of genitourinary medical oncology at the University of Texas MD Anderson Cancer Center. Rather, the interleukin-2 pathway therapies reported in the present study were exclusively modified interleukin-2–targeting drugs used in combination with ICIs in ongoing clinical trials. Our study population also included patients receiving combinations of ICIs with molecularly targeted therapies or cytotoxic chemotherapies, commonly used as standard-of-care regimens in solid tumor cancers.^22,23,24^ The inclusiveness of our eligibility criteria is intended to facilitate the generalizability of our findings toward diverse ICI-based combination regimens, which compose the majority of immunotherapies currently used or under investigation in solid tumor cancers. The distribution of adverse events observed in our study (eFigure 7 in the Supplement) reflected known rates of adverse events with ICI regimens in genitourinary cancers, with the exception of hyperglycemia, which was more frequently observed in this cohort.^25,26,27,28,29,30^

In contrast to most cytotoxic chemotherapy, but similar to molecularly targeted therapies, ICI-associated toxic effects are more diverse and occur remote to therapy with great variability in time to manifestation.^1,2,3^ Our electronic approach supports care specific to individual patients experiencing novel toxicity patterns with which the care team had limited initial familiarity. The aggregate experience across the entire patient-care team cohort allowed us to screen for meaningful, unanticipated patterns, which led to changes in our overall approach. For example, although muscle pain is a known symptom of myositis, our data revealed it to be a nonspecific precursor of this toxicity. Furthermore, although shortness of breath was associated with pulmonary toxicity requiring intervention, cough was not. These observations led to data-informed corrections in our symptom alert thresholds to prevent noninformative interactions, underscoring the merits of continuously monitoring our design’s performance.

A central goal of our study was to efficiently determine when a reported symptom required prompt action. Among symptoms that triggered alerts, 7% were linked to a treatment course correction that included advice to withhold therapy, adjust dosing, initiate a supportive remedy, or undergo emergency center evaluation. In contrast, when no alert was triggered, 95% of the time no related excess toxicity or intervention was seen. The use of a specific and objective definition of appropriate alerts allowed us to prioritize sensitivity while dynamically adapting our symptom alert thresholds to reduce the burden of unnecessary alerts to the care teams. We focused on preserving sensitivity, reasoning that it is preferable for symptom alerts to occasionally trigger clinical review even if an intervention is not ultimately needed. For this reason, we increased our alert threshold stringency only if this change would lead to an increase in negative predictive value without decreasing sensitivity.

The technology developed was well embraced by care teams and patients, resulting in sustained use with 100% of patients using the platform every month during their treatment. No patients who were approached and eligible to enroll in the protocol declined to participate. This high acceptability may be facilitated by the robust data security used by our platform. Despite the advanced age of many of our patients (25% older than 71 years), the phone-based technology was found to be manageable. These findings establish feasibility, unencumbered alignment with traditional clinical workflow, and robustness of data. After the present interim analysis demonstrated its feasibility in the first 50 patients enrolled, the platform has now been expanded beyond the department of genitourinary medical oncology to other MD Anderson Houston area locations, and for use in other indications, such as clinical trials of novel inpatient cellular immunotherapies for genitourinary cancers. Accrual is ongoing and these analyses will be reported separately.

### Limitations

The study has limitations. It focused on short-term care decisions, and the long-term cumulative impact remains unknown. The planned future analysis will further examine use of the system measured by patient outcomes and utility as sustained efficiency to outcomes. In addition, the present report is a feasibility study of the fundamental features of our technology-enabled platform. Over time, this dynamic effort will test whether changes in the diagnostic algorithms and remedies can improve individual alerts and patient outcomes. Furthermore, our study was conducted in a single center with a higher proportion of men and patients of White race, which can bias the generalizability of the results.

## Conclusions

The findings of this study suggest that automated electronic alerts followed by prompt and specific responses to emergent immune toxic effects can be delivered at scale by our platform. The ability to electronically and accurately provide patient-reported information facilitates individualized remote patient care. Continuous monitoring of our platform’s performance at the overall population level allowed us to identify correctable inefficiencies in our approach. The principles on which the model is founded provide accurate, and necessary information to patients and their care teams and serve as a paradigm for beneficial electronically enabled remote care.

## Notes

Supplement. **eMethods.** Detailed Methods **eTable 1.** Patient Comorbidity Details Using the Elixhauser Method (N=47 Patients) **eTable 2.** Baseline Smart Phone Usage Characteristics (N=47 Patients) **eTable 3.** Symptom Duration. Length of Symptom Report Is Counted in Days to Next Report Where Symptom Is Not Present **eTable 4.** Symptom Alert Threshold Changes **eFigure 1.** CONSORT Diagram **eFigure 2.** Tukey Box Plots of Patient Compliance Rates Stratified by Sex or Age Group **eFigure 3.** Example Individual Patient Course Monitored Longitudinally for Adverse Events (AEs) After Cycle 1 Day 1 (C1D1) of Immune Checkpoint Therapy Initiation **eFigure 4.** Individual Patient Reports and Alerts Collected Over Time for the Symptoms That Did Not Require Alert Threshold Updating **eFigure 5.** Individual Patient Reports and Alerts for Each Symptom Collected Over Time **eFigure 6.** Duration of Symptoms **eFigure 7.** Distribution of Grade 1-2 and Grade 3+ Adverse Events That Occurred in Patients While on Study **eFigure 8.** Funnel Plot Detailing Data Collected at Each Step of Symptom Reporting and Alerting **eAppendix 1.** Screenshots of the Patient Mobile Application for iOS and Android Devices and of the Dashboard for Clinicians **eAppendix 2.** Survey Questions and Prespecified Alert Thresholds **eAppendix 3.** Standardized Algorithms for ICI Toxicity Evaluation and Management **eAppendix 4.** Representative Quotes From Telephone Encounter Notes **eAppendix 5.** Formulas Used to Calculate the Positive Predictive Value (PPV), Negative Predictive Value (NPV), Sensitivity, and Specificity of the Symptom Alert Thresholds
eReferences
