# Supplementary material for: Evaluation of Technology-Enabled Monitoring of Patient-Reported Outcomes to Detect and Treat Toxic Effects Linked to Immune Checkpoint Inhibitors
Source: JAMA Netw Open. 2021 Aug 30;4(8):e2122998. doi: 10.1001/jamanetworkopen.2021.22998 (PMC8406081; doi:10.1001/jamanetworkopen.2021.22998)
Supplement: Supplement. — eMethods. Detailed Methods eTable 1. Patient Comorbidity Details Using the Elixhauser Method (N=47 Patients) eTable 2. Baseline Smart Phone Usage Characteristics (N=47 Patients) eTable 3. Symptom Duration. Length of Symptom Report Is Counted in Days to Next Report Where Symptom Is Not Present eTable 4. Symptom Alert Threshold Changes eFigure 1. CONSORT Diagram eFigure 2. Tukey Box Plots of Patient Compliance Rates Stratified by Sex or Age Group eFigure 3. Example Individual Patient Course Monitored Longitudinally for Adverse Events (AEs) After Cycle 1 Day 1 (C1D1) of Immune Checkpoint Therapy Initiation eFigure 4. Individual Patient Reports and Alerts Collected Over Time for the Symptoms That Did Not Require Alert Threshold Updating eFigure 5. Individual Patient Reports and Alerts for Each Symptom Collected Over Time eFigure 6. Duration of Symptoms eFigure 7. Distribution of Grade 1-2 and Grade 3+ Adverse Events That Occurred in Patients While on Study eFigure 8. Funnel Plot Detailing Data Collected at Each Step of Symptom Reporting and Alerting eAppendix 1. Screenshots of the Patient Mobile Application for iOS and Android Devices and of the Dashboard for Clinicians eAppendix 2. Survey Questions and Prespecified Alert Thresholds eAppendix 3. Standardized Algorithms for ICI Toxicity Evaluation and Management eAppendix 4. Representative Quotes From Telephone Encounter Notes eAppendix 5. Formulas Used to Calculate the Positive Predictive Value (PPV), Negative Predictive Value (NPV), Sensitivity, and Specificity of the Symptom Alert Thresholds eReferences [file jamanetwopen-e2122998-s001.pdf]

Supplementary Online Content

Msaouel P, Oromendia C, Siefker-Radtke AO, et al. Evaluation of technology-enabled monitoring of patient-reported outcomes to detect and treat toxic effects linked to immune checkpoint inhibitors. *JAMA Netw Open*. 2021;4(8):e2122998. doi:10.1001/jamanetworkopen.2021.22998

eMethods. Detailed Methods

eTable 1. Patient Comorbidity Details Using the Elixhauser Method (N=47 Patients)

eTable 2. Baseline Smart Phone Usage Characteristics (N=47 Patients)

eTable 3. Symptom Duration. Length of Symptom Report Is Counted in Days to Next Report Where Symptom Is Not Present

eTable 4. Symptom Alert Threshold Changes

eFigure 1. CONSORT Diagram

eFigure 2. Tukey Box Plots of Patient Compliance Rates Stratified by Sex or Age Group

eFigure 3. Example Individual Patient Course Monitored Longitudinally for Adverse Events (AEs) After Cycle 1 Day 1 (C1D1) of Immune Checkpoint Therapy Initiation

eFigure 4. Individual Patient Reports and Alerts Collected Over Time for the Symptoms That Did Not Require Alert Threshold Updating

eFigure 5. Individual Patient Reports and Alerts for Each Symptom Collected Over Time

eFigure 6. Duration of Symptoms

eFigure 7. Distribution of Grade 1-2 and Grade 3+ Adverse Events That Occurred in Patients While on Study

eFigure 8. Funnel Plot Detailing Data Collected at Each Step of Symptom Reporting and Alerting

eAppendix 1. Screenshots of the Patient Mobile Application for iOS and Android Devices and of the Dashboard for Clinicians

eAppendix 2. Survey Questions and Prespecified Alert Thresholds

eAppendix 3. Standardized Algorithms for ICI Toxicity Evaluation and Management

eAppendix 4. Representative Quotes From Telephone Encounter Notes

eAppendix 5. Formulas Used to Calculate the Positive Predictive Value (PPV), Negative Predictive Value (NPV), Sensitivity, and Specificity of the Symptom Alert Thresholds

eReferences

This supplementary material has been provided by the authors to give readers additional information about their work.

© 2021 Msaouel P et al. *JAMA Network Open.*

eMETHODS: Detailed Methods

Patient eligibility

Patients were eligible if they were ≥ 18 years of age, able to provide informed consent, had Eastern Cooperative Group (ECOG) performance status ≤ 3, had a personal smartphone with either iOS or Android operating systems, were fluent in English, and were commencing any line of outpatient ICIs in the MDACC Genitourinary Cancer Center or MDACC clinics in the Houston area as standard of care or as part of therapeutic clinical trial protocols. The study focused on genitourinary malignancies because only these patients are seen at the MDACC department of genitourinary medical oncology where the electronic platform was originally developed. Eligible patients could have any genitourinary malignancy and planned to start any immunotherapy regimen that included an ICI alone or in combination with other ICIs, immunotherapies, cytotoxic chemotherapy, or molecularly targeted therapies such as tyrosine kinase inhibitors. The race of each participant was assessed to determine the representativeness of the sample population. Patients self-reported their race.

Electronic infrastructure

Beyond the content of the questions and thresholds for care team notification, a fit-for-purpose platform was needed to enable active patient and care team participation. A mobile application was designed for ease of use in this patient population, with large buttons and clear font, in both iOS and Android operating systems (e**Appendix B**). The application notified patients three times per week to submit symptom reports, and once

© 2021 Msaouel P et al. *JAMA Network Open.*

completed the embedded algorithm displayed a summary. Patients could also submit symptoms at any point, if desired. If any of the questions met the severity thresholds previously determined, the patient would be prompted to contact their care team with a direct link to phone them.

The MDACC clinical trials platform Prometheus was leveraged as the central source of care team notifications for this trial. A dashboard was created to display summaries of patient reports of alertable symptoms, which were also emailed to their care teams in real time (e**Appendix C**). Care teams were instructed to review and resolve these alerts within three days of occurrence by indicating their response (contacted patient, patient went to ER, no follow-up needed). The cancer treatment of all patients enrolled in the protocol was monitored by each patient's medical oncologist and their respective care teams (advanced practice provider and clinical nurse). Patients were monitored between visits via a smartphone application downloaded onto the patient's personal device. All data resided on the secure, password-protected, 21CFR part 11–compliant Prometheus database at MDACC. Data stored on the mobile app and transferred between systems were similarly protected by secure transmission protocols and HIPAA-compliant databases. All communication between the app and other systems used HTTPS. All patient information was secured using encryption on the mobile device, which for Android devices consisted of the Android 128 bit AES(GCM), and for iOS consisted of Apple's Keychain Encryption (AES-256-GCM).

© 2021 Msaouel P et al. *JAMA Network Open.*

Methodology applied to identify the set of electronic questions and their initial alert thresholds

The set of symptoms and associated questions that was created to detect upcoming adverse events is included in e**Appendix C**. A team of clinical experts identified common and/or severe toxicities among this patient group, as well as their corresponding clinical symptoms. Having selected the appropriate symptoms, questions for each were created to capture necessary details to understand when immediate interaction of patients with their care teams was necessary. The PRO-CTCAE[1] provided inspiration but has not been validated for immunotoxicity monitoring nor high-frequency electronic reporting.[2-4]

To achieve high patient compliance, a team inclusive of clinical content experts, statisticians, user experience experts, and designers arrived at a final set of queries to assure efficiency without compromising goals. First, all symptom survey questions were reviewed, language simplified and shortened whenever possible, and parallel structures maintained across questions to aid comprehension.[5] Second, an initial multiple-choice question was created in which patients selected which symptoms they were experiencing. This allowed patients to only answer a small subset of questions, i.e., those relevant to their current state.

Uniform and precisely defined symptom queries were central to the study. Symptomatic patients selected from a tailored inventory to inform the care team of toxicity details in a standardized format. The nature and severity of the reported toxicity was used to prompt

© 2021 Msaouel P et al. *JAMA Network Open.*

alerts and recommend consensus remedies (e**Appendix D**). The initial thresholds for these alerts (e**Appendix C**) were set based on clinician consensus (e.g., reporting of severe or very severe cough) or a state known to lead to a severe symptom within two weeks (e.g., reporting of fever). This heightened level of granularity and frequency of symptom collection is not often available. Thus, dynamic adaptation of these alert thresholds was planned as described below.

To allow for unanticipated patient concerns, provisions were made to encourage patients to write in any additional symptoms in free text form. All text entries triggered an alert for care teams to review, as clinical severity could not be determined in real time.

Data collection

Patient answers and care team responses were collected in real time and monitored weekly by trial staff. Research teams also graded adverse events using CTCAE guidelines[6] via chart review every four to six weeks. A monthly cadence was used to extract dates and details of clinical interventions (defined above). Assignment of interventions to adverse events was manually adjudicated by the principal investigator while blinded to concurrent symptom responses.

© 2021 Msaouel P et al. *JAMA Network Open.*

Consensus events meriting alerts

The goal of the monitoring system was to alert the care team to acute, clinically meaningful toxicities. We defined these adverse events as those with Common Terminology Criteria for Adverse Events (CTCAE) grade of at least 2 and that were associated with a clinical intervention. Clinical interventions were defined as treatment modifications (pause, dose reduction, or termination of immunotherapy, concomitant targeted therapy, or supportive therapy) or new interactions with healthcare providers due to toxicities that developed after treatment initiation, including emergency department visits or hospitalizations. Adverse events within two weeks of an alert were considered temporally *linked* to that alert.

The symptoms included in this study were those judged by the panel of experts, described below, to be of significant concern to warrant alerting care teams and patients for potential intervention. The electronically enabled alerts were considered appropriate when they were followed by a linked intervention as defined above. Although beyond the scope of this initial report, effective alerts were those that led to remedies that mitigated toxicity. Robust analysis of effectiveness, using this definition, requires longer follow-up and will be the subject of a subsequent report.

Dynamic alert thresholds

Operating characteristics were estimated for each symptom alert and were conditional on the symptom having been reported. To prevent over-indexing for patients with more alerts, subsequent reports for a patient were downweighted by a factor of 1/k, where k is

© 2021 Msaouel P et al. *JAMA Network Open.*

the number of alerts [for positive predictive value (PPV) and sensitivity] or non-alerts [for negative predictive value (NPV) and specificity] for that symptom for that patient. The formulas used for PPV, NPV, sensitivity, and specificity are listed in e**Appendix F**.

The analysis included stringent requirements for threshold increases to ensure patient safety, reduce risk of overfitting, and account for the fact that a single symptom alert could be triggered by one of several associated questions. Symptoms with a sensitivity smaller than 10% were identified as candidates for an increase in threshold stringency. Within each symptom, questions that had led to alerts in at least five unique patients were considered, with those more commonly alerting studied first. The impact of increasing this initial question's alert threshold by one level was studied by calculating the NPV and sensitivity of the full symptom alert under this counterfactual condition. The change was recommended if the NPV increased and the sensitivity did not decrease (i.e., none of the alerts removed were linked with subsequent adverse events). If this change was found to be beneficial, the subsequent level was considered, as well as the next most common alerting question for that symptom. This procedure continued until a question did not lead to a change, at which point no further questions were considered for that symptom and the analysis proceeded to the next symptom. Recommended changes were then reviewed and approved by clinical experts before implementation.

© 2021 Msaouel P et al. *JAMA Network Open.*

eTable 1. Patient comorbidity details using the Elixhauser method (N=47 patients).

**Hypertension, uncomplicated, n (%)**	32 (68%)
**Diabetes, complicated, n (%)**	5 (11%)
**Hypothyroidism, n (%)**	11 (23%)
**Metastatic cancer, n (%)**	39 (83%)
**Solid tumor, n (%)**	47 (100%)
**Fluid and electrolyte disorders, n (%)**	9 (19%)
**Weight loss, n (%)**	7 (15%)
**Renal failure, n (%)**	21 (45%)
**Coagulopathy, n (%)**	3 (6.4%)
**Cardiac arrhythmias, n (%)**	13 (28%)
**Peripheral vascular disorders, n (%)**	5 (11%)
**Rheumatoid arthritis/collagen vascular disease, n (%)**	2 (4.3%)
**Congestive heart failure, n (%)**	2 (4.3%)
**Pulmonary circulation disorders, n (%)**	1 (2.1%)
**Chronic pulmonary disease, n (%)**	2 (4.3%)
**Hypertension, complicated, n (%)**	2 (4.3%)
**Peptic ulcer disease, excluding bleeding, n (%)**	1 (2.1%)
**Other neurological disorders, n (%)**	1 (2.1%)
**Liver disease, n (%)**	2 (4.3%)
**Diabetes, uncomplicated, n (%)**	1 (2.1%)
**Depression, n (%)**	3 (6.4%)
**Obesity, n (%)**	1 (2.1%)
**Valvular disease, n (%)**	1 (2.1%)

© 2021 Msaouel P et al. *JAMA Network Open.*

eTable 2. Baseline smart phone usage characteristics (N=47 patients).

Topic	Question	Answer	Number of Patients	% Patients
Access to Help on Computer	Do you have someone you can ask for help if you have any questions relating to computers?	Yes	41	87.23%
		No	6	12.77%
Text Confidence	How confident are you text messaging using your smart phone?	Not confident at all	0	0.00%
		I usually need help	6	12.77%
		It depends on the task	3	6.38%
		Confident	38	80.85%
Internet Confidence	How confident are you using an internet browser on your smart phone?	Not confident at all	3	6.38%
		I usually need help	8	17.02%
		It depends on the task	12	25.53%
		Confident	24	51.06%
App Confidence	How confident are you using apps on your smart phone?	Not confident at all	0	0.00%
		I usually need help	12	25.53%
		It depends on the task	11	23.40%
		Confident	24	51.06%
Phone Confidence	How confident are you with a smart phone?	Not confident at all	2	4.26%
		I usually need help	8	17.02%
		It depends on the task	13	27.66%
		Confident	24	51.06%

© 2021 Msaouel P et al. *JAMA Network Open.*

Text Frequency	How often do you use text messaging?	Never	0	0.00%
		Monthly	1	2.13%
		Weekly	3	6.38%
		Daily	43	91.49%
App Frequency	How often do you use your smart phone apps?	Never	4	8.51%
		Monthly	5	10.64%
		Weekly	7	14.89%
		Daily	31	65.96%
Web Frequency	How often do you use your smart phone to surf the web?	Never	5	10.64%
		Monthly	3	6.38%
		Weekly	8	17.02%
		Daily	31	65.96%
Phone Skill	How would you rate your smart phone skill level?	Never used a smartphone	2	4.26%
		Beginner	16	34.04%
		Competent	29	61.70%

© 2021 Msaouel P et al. *JAMA Network Open.*

eTable 3. Symptom duration. Length of symptom report is counted in days to next report where symptom is not present.		
Abdominal Pain	Median (IQR)	6 (3, 10)
	Range	0, 69
Anxiety	Median (IQR)	4 (2, 12)
	Range	0, 167
Arthralgia/Myalgia	Median (IQR)	4 (2, 8)
	Range	0, 151
Blurry Vision	Median (IQR)	6 (2, 18)
	Range	0, 143
Concentration/Memory Issues	Median (IQR)	3.5 (1.8, 6.0)
	Range	0.0, 15.0
Cough	Median (IQR)	3 (2, 9)
	Range	0, 85
Diarrhea	Median (IQR)	4 (2, 9)
	Range	0, 240
Dizziness	Median (IQR)	4.0 (2.0, 8.0)
	Range	0.0, 34.0
Dysuria	Median (IQR)	5.0 (3.2, 7.5)
	Range	2.0, 22.0
Fatigue	Median (IQR)	5 (2, 13)
	Range	0, 189
Fever	Median (IQR)	3.0 (2.0, 4.0)

© 2021 Msaouel P et al. *JAMA Network Open.*

	Range	0.0, 13.0
Nausea/Vomiting	Median (IQR)	3.5 (2.0, 8.8)
	Range	0.0, 36.0
Pain	Median (IQR)	6 (2, 16)
	Range	0, 82
Palpitations	Median (IQR)	3.00 (2.00, 4.00)
	Range	1.00, 7.00
Pruritus	Median (IQR)	7 (3, 15)
	Range	0, 89
Shortness of Breath	Median (IQR)	4 (2, 6)
	Range	0, 90

© 2021 Msaouel P et al. *JAMA Network Open.*

eTable 4. Symptom alert threshold changes. Alerts were removed for the answers noted in red.

Symptom	Survey Question	Do not trigger alerts if:	Trigger alerts if:
Cough	Since your last report, is coughing a new problem for you?	No	Yes
	Please rate the severity of your cough at its worst:	Mild; Moderate; Severe	~~Severe;~~ Very Severe
	Is the severity of your cough getting worse?	No	Yes
	How much has the cough interfered with your usual or daily activities?	Not at all; A little bit; Somewhat	Quite a bit; Very much
Arthralgia / Myalgia	Since your last report, have you had aching muscles?	Yes; No	
	Are aching muscles a new problem for you?	No; Yes	~~Yes~~
	Please rate the severity of your aching muscles at its worst:	Mild; Moderate	Severe; Very severe
	How often have you had aching muscles?	Rarely; Occasionally; Frequently	~~Frequently;~~ Almost Constantly
	How much have your aching muscles interfered with your usual or daily activities?	Not at all; A little bit; Somewhat; Quite a bit	~~Quite a bit;~~ Very much
	Since your last report, have you had aching joints (such as elbows, knees, shoulders)?	Yes; No	
	Are aching joints (such as elbows, knees, shoulders) a new problem for you?	No; Yes	~~Yes~~
	Please rate the severity of your aching joints (such as elbows, knees, shoulders) at its worst:	Mild; Moderate	Severe; Very severe

© 2021 Msaouel P et al. *JAMA Network Open.*

	How often have you had aching joints (such as elbows, knees, shoulders)?	Rarely; Occasionally; Frequently	Frequently; Almost Constantly
	How much have your aching joints (such as elbows, knees, shoulders) interfered with your usual or daily activities?	Not at all; A little bit; Somewhat; Quite a bit	Very much
Fatigue	Please rate the severity of your fatigue, tiredness, or lack of energy at its worst:	Mild; Moderate	Severe; Very severe
	How much has your fatigue, tiredness, or lack of energy interfered with your usual or daily activities?	Not at all; A little bit; Somewhat; Quite a bit; Very much	Quite a bit; Very much

© 2021 Msaouel P et al. *JAMA Network Open.*

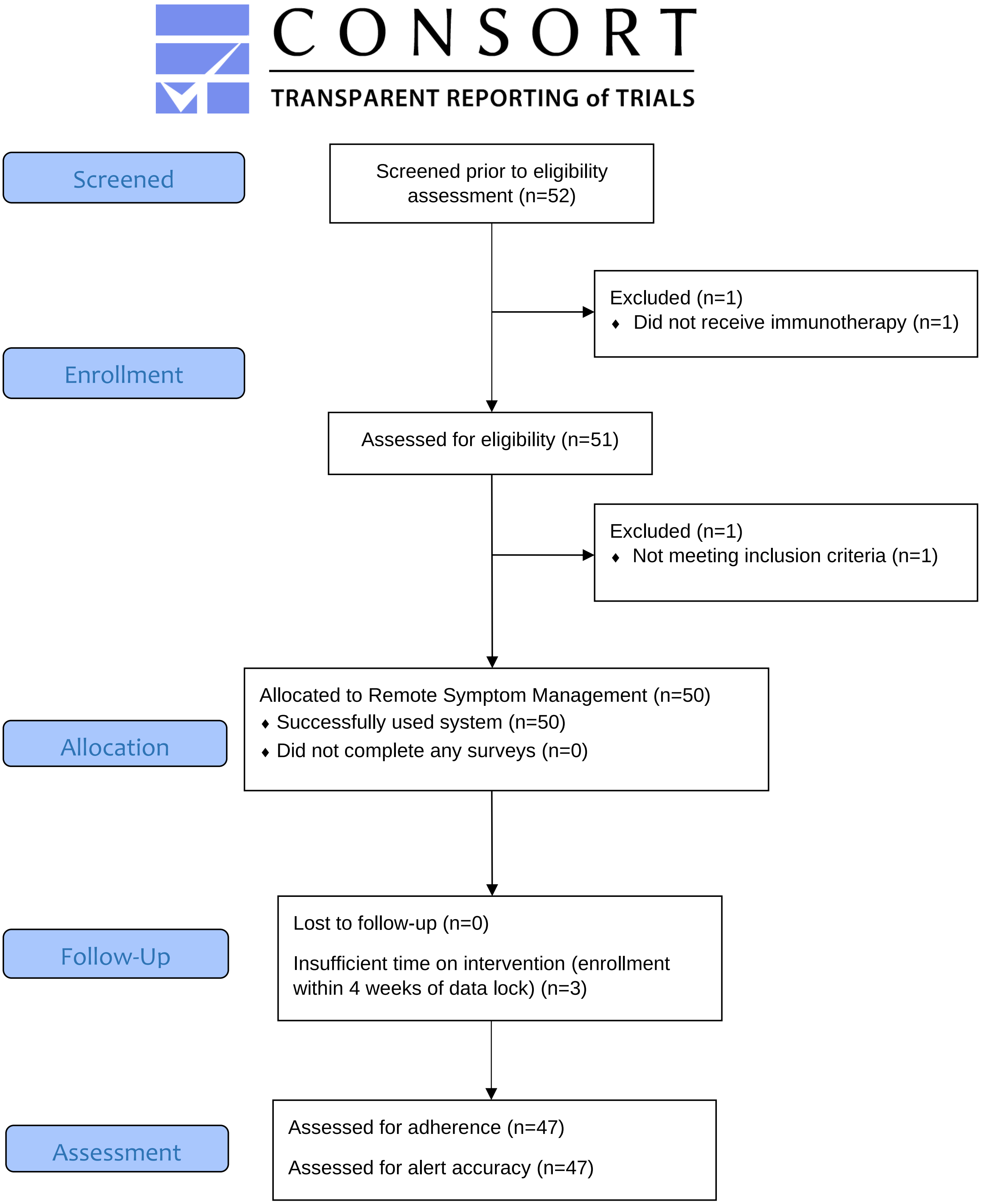

eFigure 1. CONSORT diagram.

© 2021 Msaouel P et al. *JAMA Network Open.*

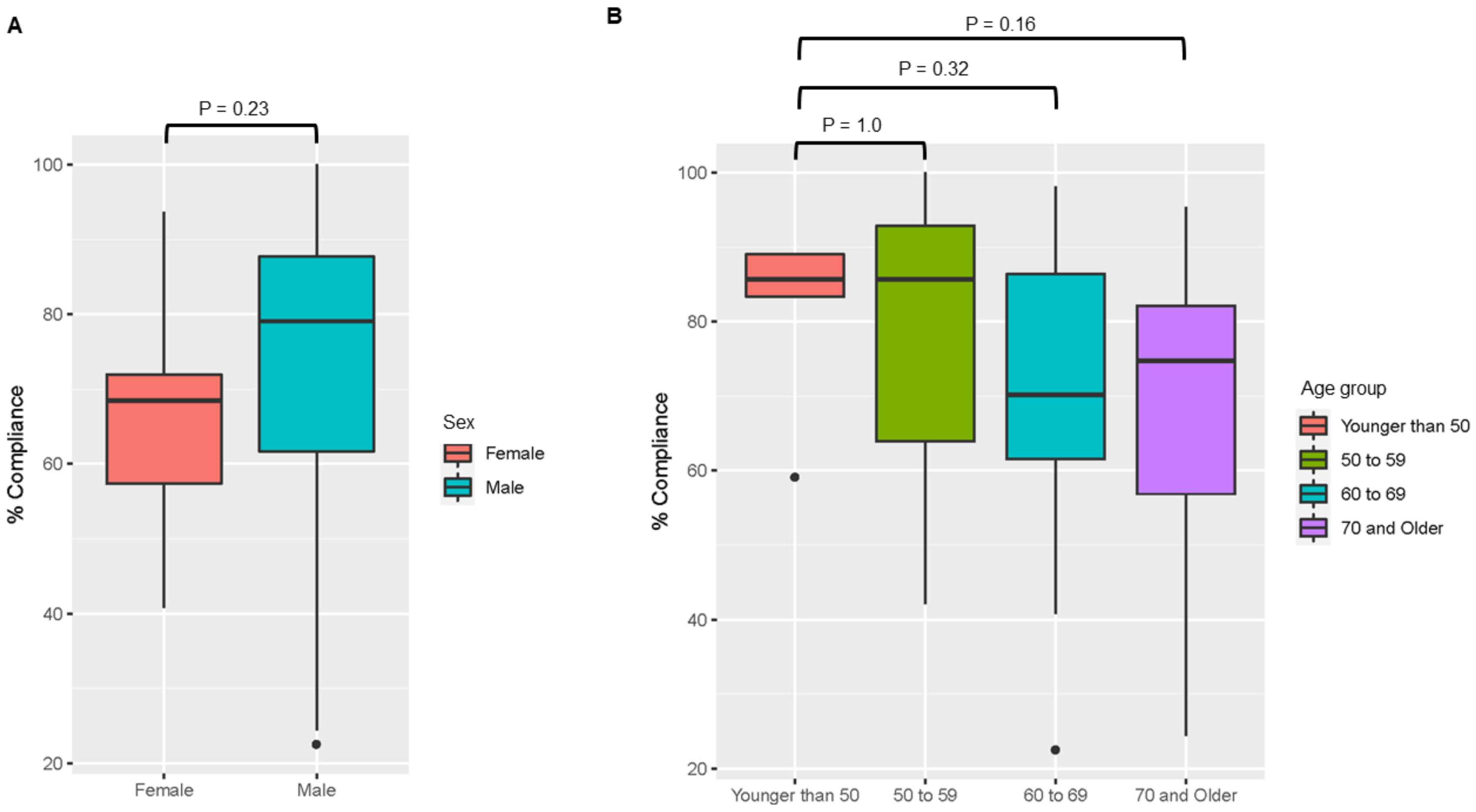

eFigure 2. Tukey box plots of patient compliance rates stratified by **(A)** sex or **(B)** age group. For each box plot, the central rectangle spans the interquartile range (IQR), the segment within the rectangle shows the median, and the upper and lower whiskers respectively extend the upper and lower hinges of the rectangle by 1.5× IQR. Black dots represent outliers outside 1.5× IQR from each hinge. Comparisons between groups were performed using Mann-Whitney U tests.

© 2021 Msaouel P et al. *JAMA Network Open.*

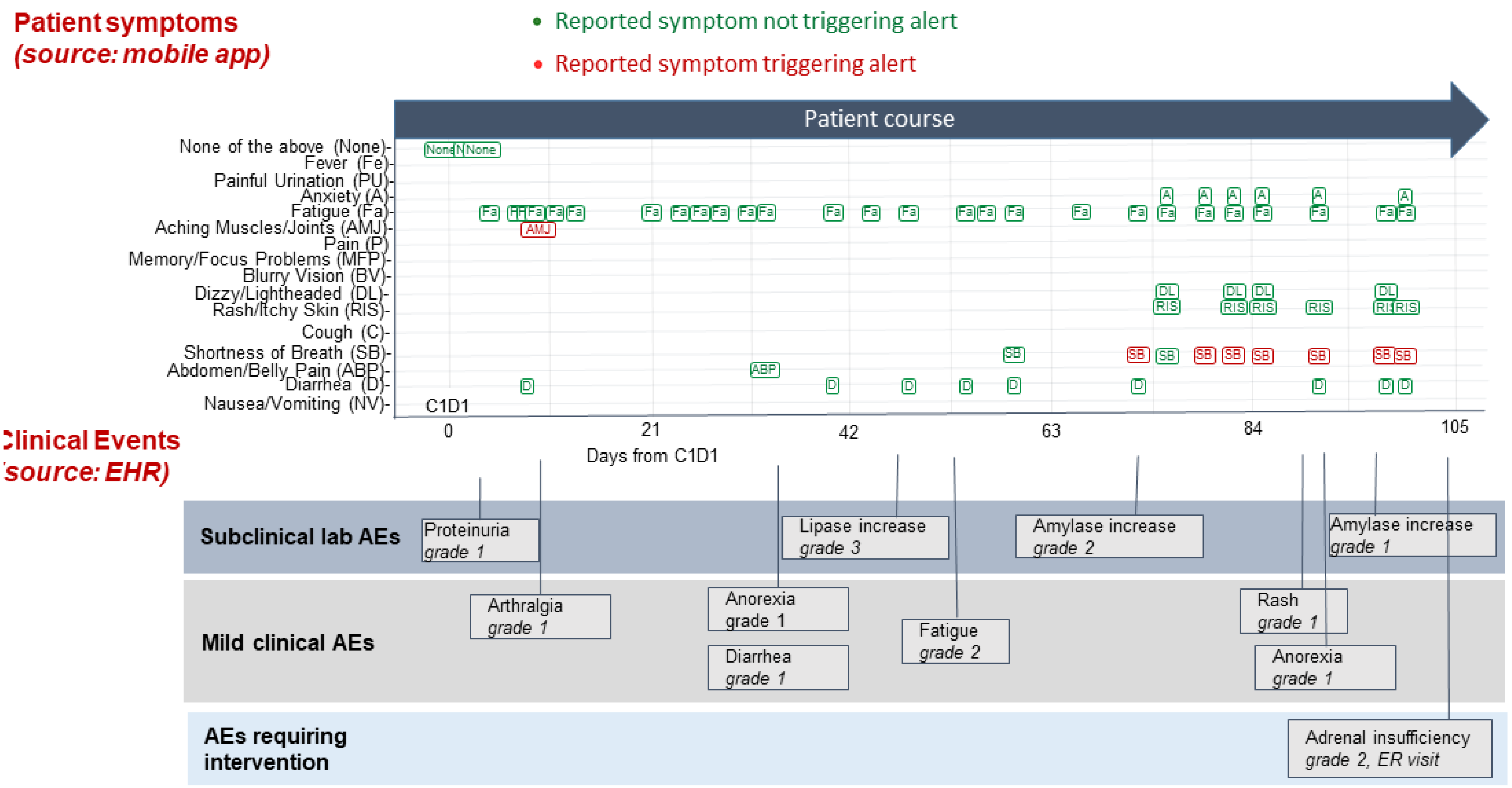

eFigure 3. Example Individual Patient Course Monitored Longitudinally for Adverse Events (AEs) After Cycle 1 Day 1 (C1D1) of Immune Checkpoint Therapy Initiation. Key milestones that were electronically identified and informed course corrections are shown. Electronically reported symptoms in green did not trigger alerts, whereas those in red triggered alerts. The patient was monitored using the originally chosen alert thresholds. The early alert triggered for muscle pain was not associated with any clinically meaningful AEs requiring intervention. The alert triggered for shortness of breath preceded the immune-related AE by more than 2 weeks prior to the ER visit that led to the diagnosis of adrenal insufficiency. The symptom resolved following adrenal steroid replacement therapy.

© 2021 Msaouel P et al. *JAMA Network Open.*

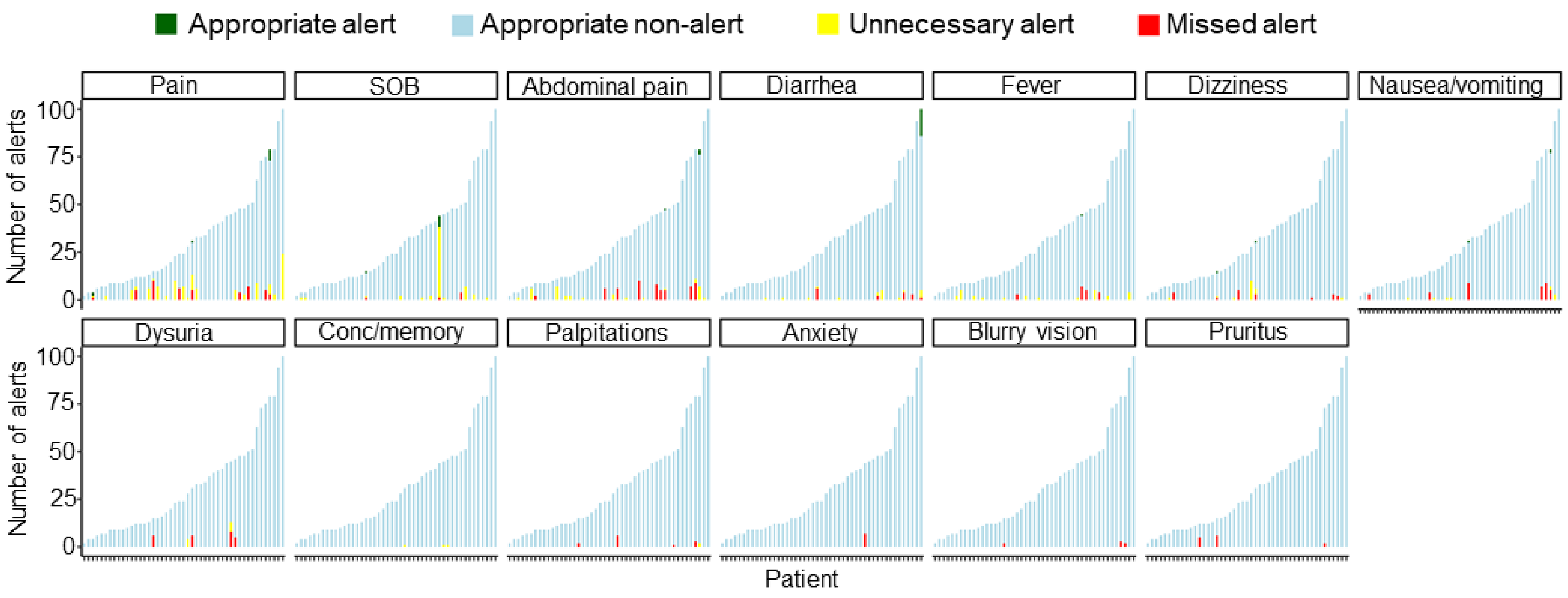

eFigure 4. Individual patient reports and alerts collected over time for the symptoms that did not require alert threshold updating. Appropriate alerts were those linked to clinical interventions. Appropriate non-alerts were defined as the absence of prompts following symptom reports that were not linked to clinical interventions. Unnecessary alerts were alerts following symptom reports that were not linked to clinical interventions. Missed alerts were defined as the absence of prompts following symptom reports that were linked to clinical interventions. Conc/memory: Concentration/Memory issues; SOB: shortness of breath.

© 2021 Msaouel P et al. *JAMA Network Open.*

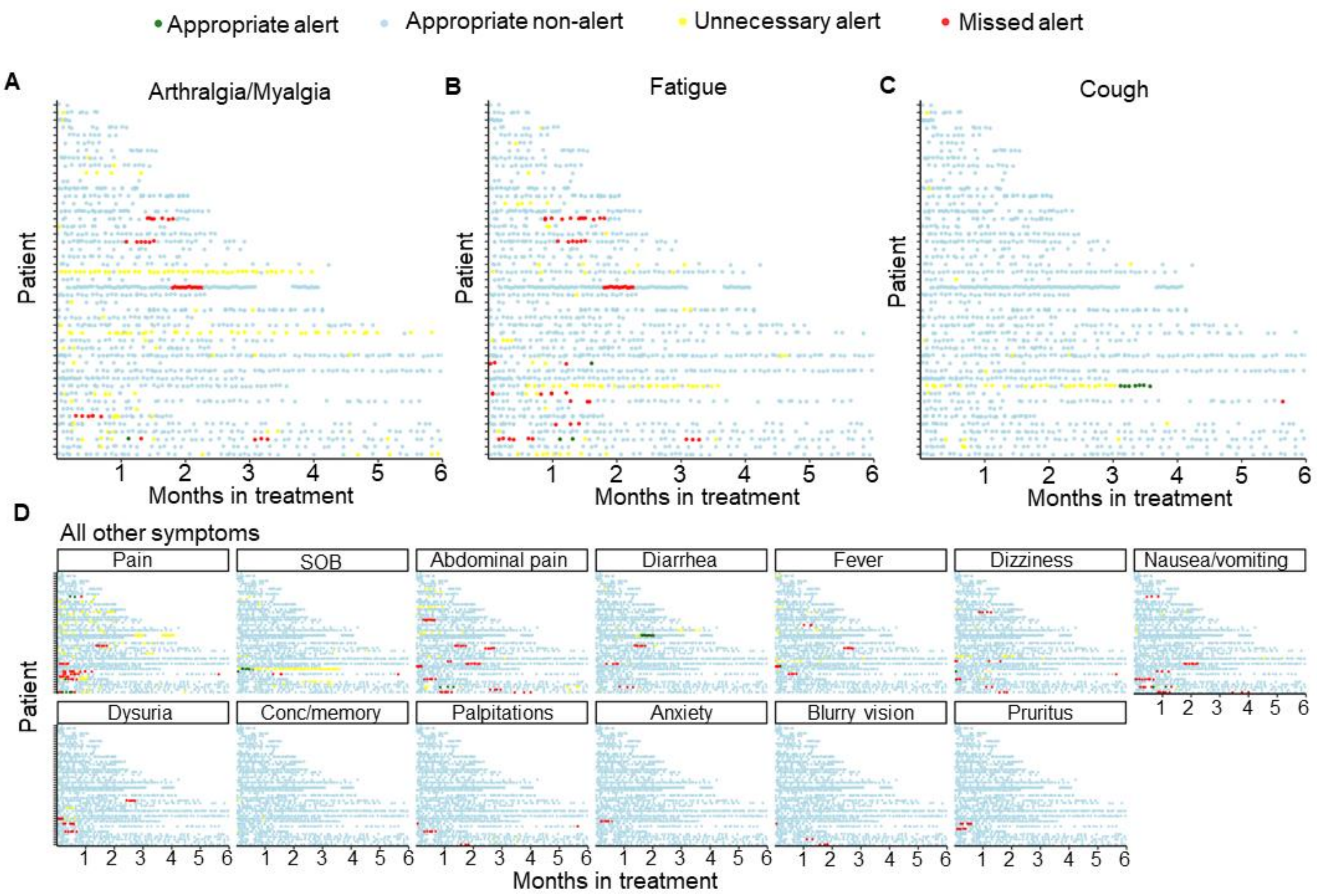

eFigure 5. Individual patient reports and alerts for each symptom collected over time. **(A-C)** Symptoms that required alert threshold updating. **(D)** All other symptoms. Appropriate alerts were those linked to clinical interventions. Appropriate non-alerts were defined as the absence of prompts following symptom reports that were not linked to clinical interventions. Unnecessary alerts were alerts following symptom reports that were not linked to clinical interventions. Missed alerts were defined as the absence of prompts following symptom reports that were linked to clinical interventions. Conc/memory: Concentration/Memory issues; SOB: shortness of breath.

© 2021 Msaouel P et al. *JAMA Network Open.*

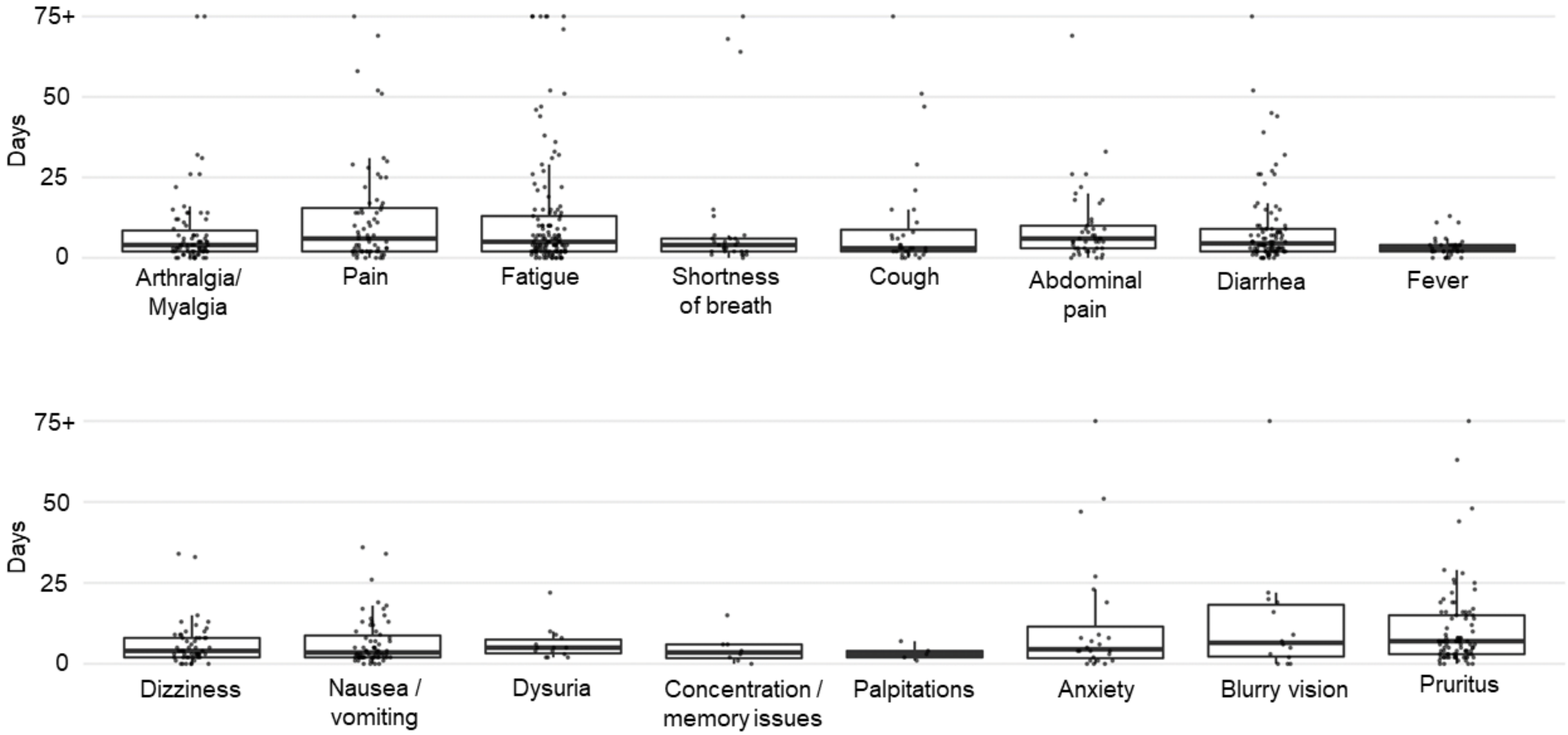

eFigure 6. Duration of symptoms. Length of symptom report is counted in days to next report where symptom is not present. For each box plot, the central rectangle spans the interquartile range (IQR), the segment within the rectangle shows the median, and the upper and lower whiskers respectively extend the upper and lower hinges of the rectangle by 1.5× IQR.

© 2021 Msaouel P et al. *JAMA Network Open.*

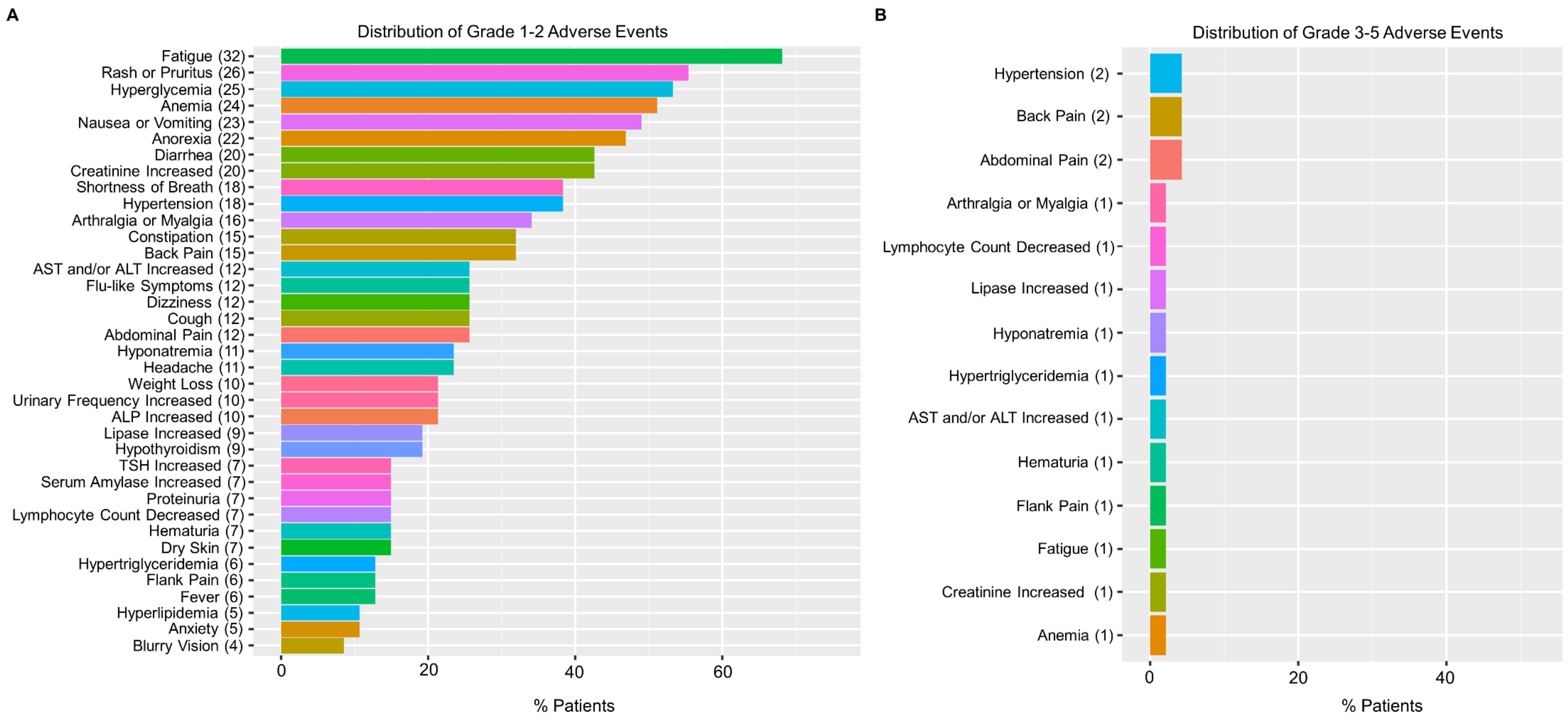

eFigure 7. Distribution of grade 1-2 **(A)** and Grade 3+ **(B)** adverse events that occurred in patients while on study. CTCAE grading was used. Numbers in parenthesis correspond to the absolute numbers of patients that experienced each adverse event.

© 2021 Msaouel P et al. *JAMA Network Open.*

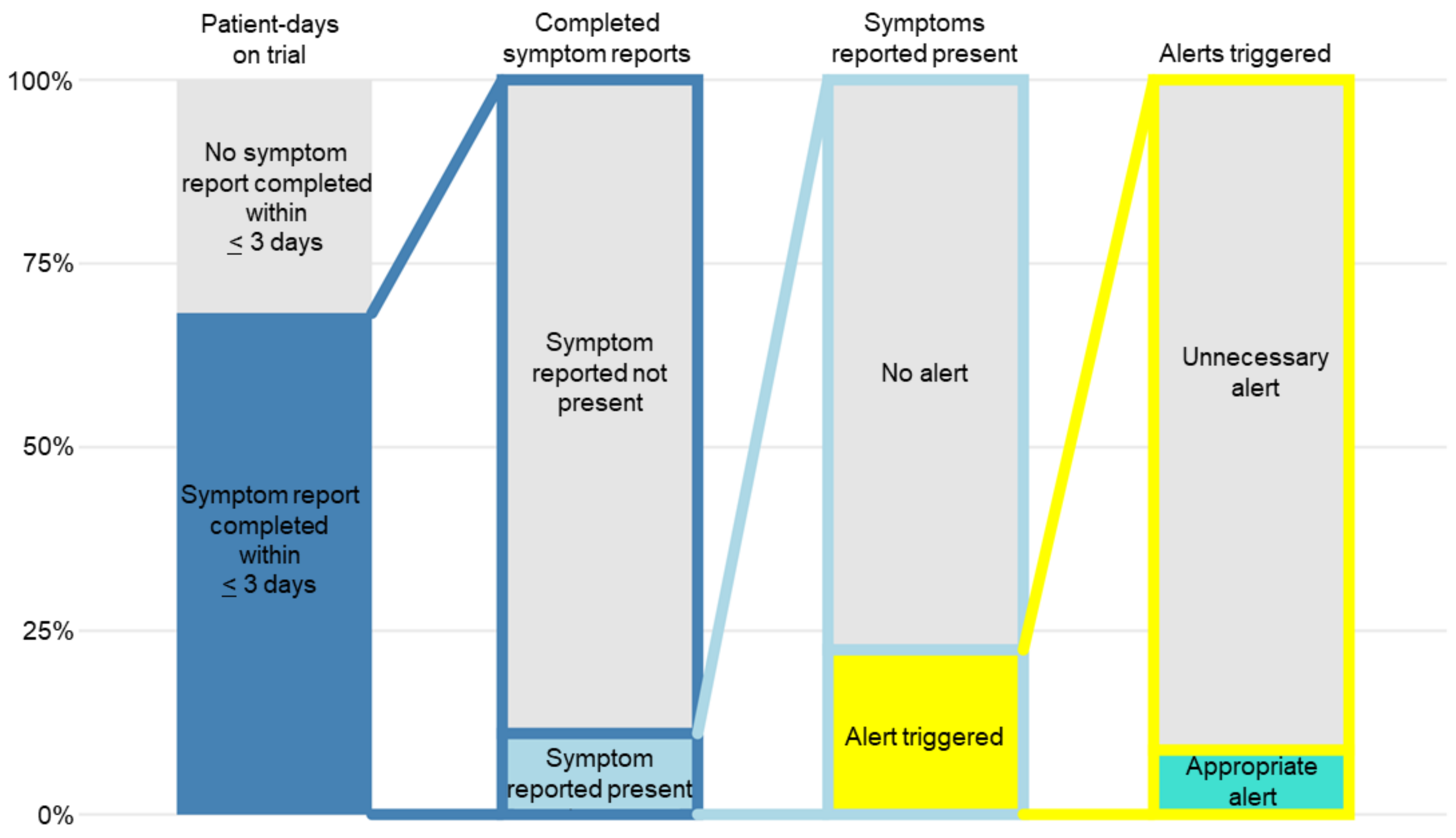

eFigure 8. Funnel plot detailing data collected at each step of symptom reporting and alerting.

© 2021 Msaouel P et al. *JAMA Network Open.*

eAppendix 1. Screenshots of the patient mobile application for iOS and Android devices and of the dashboard for clinicians.

iOS Mobile Application

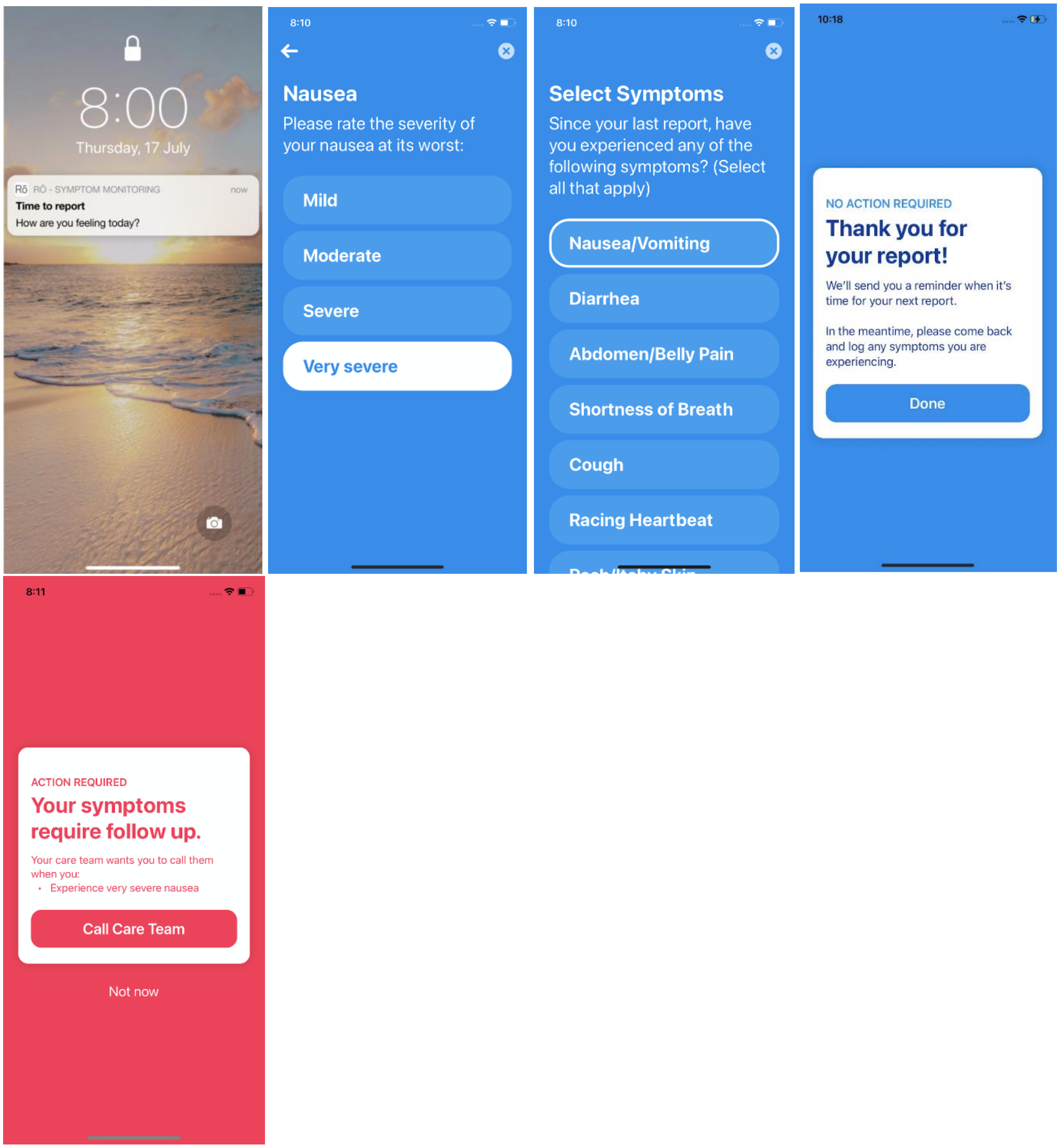

© 2021 Msaouel P et al. *JAMA Network Open.*

eAppendix 2. Survey questions and pre-specified alert thresholds.

Symptom	Survey Question	Do not trigger alerts if:	Trigger alerts if:
Nausea / Vomiting	Please rate the severity of your nausea at its worst:	Mild; Moderate	Severe; Very severe
	How often have you been nauseous?	Rarely; Occasionally; Often	Frequently; Almost constantly
	Since your last report, have you vomited?	Yes; No	
	Please rate the severity of your vomiting at its worst:	Mild; Moderate	Severe; Very severe
	How many times per day did you vomit?	0; 1-2	3-4; 5+
	Have you had trouble keeping liquids down?	No	Yes
Diarrhea	On average, how many bowel movements do you have per day when you are feeling normal?	0-1; 2-4; 5+	
	How many episodes of loose or watery stools are you having per day at its worst?	0-1; 2	3+
	Do you have blood in your stool?	No	Yes
Abdominal Pain	Please rate the severity of the pain in your abdomen at its worst:	Mild; Moderate	Severe; Very severe
	How often have you had pain in your abdomen?	Rarely; Occasionally; Often	Frequently; Almost constantly
	How much has pain in your abdomen interfered with your usual or daily activities?	Not at all; A little bit	Somewhat; Quite a bit; Very much
	Has your pain in the abdomen interfered with your ability to eat or drink?	No	Yes
	Are you currently taking pain medication for your abdomen pain?	Yes; No	

© 2021 Msaouel P et al. *JAMA Network Open.*

	Is your pain medication currently controlling your pain to your satisfaction?	Yes	No
Shortness of Breath	Since your last report, is shortness of breath a new problem for you?	No	Yes
	Please rate the severity of your shortness of breath at its worst:	Mild; Moderate	Severe; Very severe
	Is the severity of your shortness of breath getting worse?	No	Yes
	How much has your shortness of breath interfered with your usual or daily activities?	Not at all; A little bit	Somewhat; Quite a bit; Very much
	Does your shortness of breath occur while you are resting?	No	Yes
Cough	Since your last report, Is coughing a new problem for you?	No	Yes
	Please rate the severity of your cough at its worst:	Mild; Moderate	Severe; Very severe
	Is the severity of your cough getting worse?	No	Yes
	How much has the cough interfered with your usual or daily activities?	Not at all; A little bit; Somewhat	Quite a bit; Very much
Palpitations	Please rate the severity of your pounding or racing heartbeat?	Mild; Moderate	Severe; Very severe
	How often do you feel a pounding or racing heartbeat?	Rarely	Occasionally; Often; Frequently; Almost constantly
Rash / Pruritus	Since your last report, have you had a rash?	Yes; No	
	Is rash a new problem for you?	No	Yes
	Please rate the severity of your rash at its worst:	Mild; Moderate	Severe; Very severe

© 2021 Msaouel P et al. *JAMA Network Open.*

	Is the severity of your rash getting worse?	No	Yes
	Since your last report, have you had itchy skin?	Yes; No	
	Please rate the severity of your itchy skin at its worst:	Mild; Moderate; Severe	Very severe
Dizziness / Lightheadedness	Since your last report, is dizziness/lightheadedness a new problem for you?	No	Yes
	How much has dizziness/lightheadedness interfered with your usual or daily activities?	Not at all; A little bit	Somewhat; Quite a bit; Very much
	Have you passed out or fallen because of your dizziness?	No	Yes
Blurry Vision	Since your last report, is blurry vision a new problem for you?	No	Yes
	Please rate the severity of your blurry vision at its worst:	Mild; Moderate	Severe; Very severe
	How much has your blurry vision interfered with your usual or daily activities?	Not at all; A little bit; Somewhat	Quite a bit; Very much
	Do you have pain, itchiness or redness of the eye(s)?	No	Yes
Concentration / Memory	Since your last report, is difficulty with concentration and/or memory a new problem for you?	No	Yes
	Please rate the severity of your difficulty with concentration and/or memory at its worst:	Mild; Moderate	Severe; Very severe
	Is the severity of your difficulty with concentration and/or memory getting worse?	No	Yes
	How much has your difficulty with concentration and/or memory interfered with your usual or daily activities?	Not at all; A little bit; Somewhat	Quite a bit; Very much

© 2021 Msaouel P et al. *JAMA Network Open.*

Pain	Please rate the severity of your pain at its worst:	Mild; Moderate	Severe; Very severe
	How often have you had pain?	Rarely; Occasionally; Often	Frequently; Almost constantly
	How much has your pain interfered with your usual or daily activities?	Not at all; A little bit; Somewhat	Quite a bit; Very much
	Are you currently taking pain medication?	Yes; No	
	Is your pain medication currently controlling your pain to your satisfaction?	Yes	No
Arthralgia / Myalgia	Since your last report, have you had aching muscles?	Yes; No	
	Are aching muscles a new problem for you?	No	Yes
	Please rate the severity of your aching muscles at its worst:	Mild; Moderate	Severe; Very severe
	How often have you had aching muscles?	Rarely; Occasionally; Often	Frequently; Almost constantly
	How much have your aching muscles interfered with your usual or daily activities?	Not at all; A little bit; Somewhat	Quite a bit; Very much
	Since your last report, have you had aching joints (such as elbows, knees, shoulders)?	Yes; No	
	Are aching joints (such as elbows, knees, shoulders) a new problem for you?	No	Yes
	Please rate the severity of your aching joints (such as elbows, knees, shoulders) at its worst:	Mild; Moderate	Severe; Very severe
	How often have you had aching joints (such as elbows, knees, shoulders)?	Rarely; Occasionally; Often	Frequently; Almost constantly
	How much have your aching joints (such as elbows, knees, shoulders)	Not at all; A little bit; Somewhat	Quite a bit; Very much

© 2021 Msaouel P et al. *JAMA Network Open.*

	interfered with your usual or daily activities?		
Fatigue	Please rate the severity of your fatigue, tiredness, or lack of energy at its worst:	Mild; Moderate	Severe; Very severe
	How much has your fatigue, tiredness, or lack of energy interfered with your usual or daily activities?	Not at all; A little bit; Somewhat	Quite a bit; Very much
Anxiety	Please rate the severity of your anxiety at its worst:	Mild; Moderate; Severe	Very severe
	How much has your anxiety interfered with your usual or daily activities?	Not at all; A little bit; Somewhat; Quite a bit	Very much
Dysuria	Since your last report, is pain or burning with urination a new problem for you?	No	Yes
	Please rate the severity of pain or burning with urination:	Mild; Moderate	Severe; Very severe
Fever	Are you having fever (temperature of 100.4+)?	No	Yes

© 2021 Msaouel P et al. *JAMA Network Open.*

eAppendix 3. Standardized algorithms for ICI toxicity evaluation and management.

Diagnostic evaluation:

For all potential immune-related adverse events send the following blood tests:

- Complete blood count (CBC) with differential, comprehensive metabolic panel (CMP)
- Thyroid stimulating hormone (TSH), free T4, total T3
- Erythrocyte sedimentation rate (ESR), C-reactive protein (CRP), cytokine 12, serum protein electrophoresis (SPEP), immunofixation electrophoresis (IFE)
- Cytokine panel 3: interferon gamma (IFNγ), interleukin 6 (IL-6), tumor necrosis factor-alpha (TNFα)
- Cytokine panel 12: IL-1 beta, IL-2, IL-2 receptor, IL-4, IL-5, IL-6, IL-8, IL-10, IL-12, IL-13, IL-17, IFNγ
- T-SPOT tuberculosis
- Hepatitis B virus (HBV) panel: hepatitis B total Ig core antibody, hepatitis B surface antigen, hepatitis B surface antibody
- Hepatitis C virus (HCV) antibody

In addition, the following tests should be sent based on specific symptoms:

For symptoms of endocrinopathy:

- Serum cortisol, adrenocorticotropic hormone (ACTH)
- Serum follicle-stimulating hormone (FSH), luteinizing hormone (LH), testosterone-M, estradiol-F

For symptoms of hypophysitis:

- Magnetic resonance imaging (MRI) brain with sella/hypopituitary focus

For symptoms of Graves' disease or thyroiditis:

- Serum thyroid peroxidase (TPO) antibody, thyroglobulin antibody, thyrotropin receptor antibody

For symptoms of pancreatitis:

- Serum amylase and lipase levels

For symptoms of hyperglycemia:

- Serum insulin levels, serum c-peptide levels, serum antibodies against glutamic acid decarboxylase (GAD65), anti-insulin, anti-islet cell A, and zinc transporter 8 (ZN-T8)

For symptoms of diarrhea/colitis:

- Stool for *Clostridium difficile* DNA polymerase chain reaction (PCR), ova and parasites, multiplex gastrointestinal pathogen panel, and cytomegalovirus (CMV)
- Stool for lactoferrin at 0, 2, and 6 months after symptom initiation

© 2021 Msaouel P et al. *JAMA Network Open.*

- Stool for calprotectin at 0, 2, and 6 months after symptom initiation (must be collected in separate container)
- Stool for fecal occult blood
- Computerized tomography (CT) of the abdomen and pelvis with and without intravenous contrast

For symptoms of hepatitis:
- Serum antinuclear antibody (ANA), immunoglobulin levels (IgG, IgM, IgA), smooth muscle antibody, mitochondrial antibody M2, antineutrophil cytoplasmic antibodies (ANCA), anti-DNA antibody (double stranded), anti-single stranded DNA (ssDNA) antibody, liver/kidney microsome type 1 antibody, tissue transglutaminase antibody IgA/IgG
- Serum ceruloplasmin, α-1-anti-trypsin, gamma-glutamyl transferase (GGT), ferritin, iron, transferrin/total iron-binding capacity (TIBC), prothrombin time (PT)/ international normalized ratio (INR)
- Serum hepatitis A virus (HAV) IgM/IgG, HBV DNA quantitation, HCV RNA detection/quantitation, hepatitis E virus (HEV) IgM/IgG, HEV RNA detection/quantitation, CMV IgM/IgG, CMV quantitative PCR, Epstein-Barr virus (EBV) antibody panel, EBV PCR, herpes simplex virus (HSV)/varicella-zoster virus (VZV) DNA, human immunodeficiency virus (HIV) 1/2 antigen/antibody
- Liver ultrasound with Doppler

For symptoms of pneumonitis:
- Sputum culture, gram stain, acid-fast bacilli (AFB), respiratory viral PCR
- Serum cardiac panel (CK-MB, troponin-T)
- Electrocardiogram, echocardiogram 2D/3D
- 6-minute walk test
- Complete pulmonary function tests
- High-resolution chest CT (pulmonary embolism protocol)

For symptoms of myositis:
- Serum creatine kinase (CK), aldolase, myositis antibody 3 panel, smooth muscle antibody, ANA, muscle-specific tyrosine kinase (MuSK) antibody
- Serum myasthenia gravis (MG)/ Lambert-Eaton syndrome (LES) panel
- Serum CK-MB, troponin T, NTproBNP, troponin I, brain natriuretic peptide (BNP)
- Electromyography (EMG)

For symptoms of myocarditis:
- EKG, chest X-ray, echocardiogram 2D/3D, cardiac MRI with and without contrast, telemetry monitoring
- Serum CK-MB, troponin T, NTproBNP, troponin I, BNP
- Serum C3, C4
- Serum CK, aldolase, myositis antibody 3 panel, smooth muscle antibody, ANA
- MG/LES panel
- EMG

For symptoms of myasthenia gravis:

© 2021 Msaouel P et al. *JAMA Network Open.*

- Serum MG/LES panel
- EMG
- Serum CK, aldolase, myositis antibody 3 panel, smooth muscle antibody, ANA
- Serum CK-MB, troponin T, NTproBNP, troponin I, BNP

For symptoms of nephritis/nephropathy:

- Urinalysis, urine protein/creatinine ratio (UPCR), urine eosinophils

Management algorithms:

The following section includes definitions of toxicity grades using Common Terminology Criteria for Adverse Events (CTCAE) version 5.0. In addition, this section contains “triggers” for initiation of management for these toxicities based on “standard” recommendations and our clinical experience.

Endocrine:

Hypothyroidism:
Grade 1: Continue immune checkpoint inhibitor (ICI) and monitor TSH, free T4
Grade 2: Continue ICI; Trigger: if mildly or moderately symptomatic or TSH > 10, then supplement thyroid hormone and consult endocrinology team
Grades 3-4: Hold ICI; Trigger: if severely symptomatic, then hospitalization, supplement thyroid hormone, and urgent consultation with endocrinology team

Hyperthyroidism:
Grade 1: Continue ICI, monitor TSH and FT4
Grade 2: Hold ICI; Tigger: if mildly or moderately symptomatic, then initiate beta blocker, consider thionamide, and consult endocrinology team
Grades 3-4: Hold ICI; Trigger: if severely symptomatic, then hospitalization, initiate beta blocker, thionamide, and steroids, and emergent consultation with endocrinology team

Hypophysitis/adrenal insufficiency:
Grade 1: Continue ICI; Trigger: if asymptomatic or mildly symptomatic, then initiate hormone replacement therapy and consult endocrinology team.
Grade 2: Continue ICI; Trigger: if moderately symptomatic, then hospitalization until stabilized on hormone replacement therapy, and urgent consultation with endocrinology team
Grades 3-4: Hold ICI; Trigger: if severely symptomatic, then hospitalization, administer stress dose corticosteroids, and emergent consultation with endocrinology team

Hyperglycemia:
Grade 1: Continue ICI; Trigger: if blood glucose between 150 and 250, then consult endocrinology team
Grade 2: Continue ICI; Trigger: if blood glucose > 250 and mildly symptomatic, then urgent consult with endocrinology team

© 2021 Msaouel P et al. *JAMA Network Open.*

Grades 3-4: Hold ICI; Trigger: if blood glucose > 250 and moderately or severely symptomatic or diabetic ketoacidosis, then hospitalization and emergent consult with endocrinology team

Hematologic:

Anemia:
Grade 1: Continue ICI; Trigger: if hemoglobin ≥ 10, then close monitoring
Grade 2: Continue ICI; Trigger: if hemoglobin 8 to 10, then close monitoring and consider consulting benign hematology team
Grades 3-4: Discontinue ICI; Trigger: if hemoglobin < 8, then urgent consult with benign hematology team.

Thrombocytopenia:
Grade 1: Continue ICI; Trigger: if platelet count ≥ 75,000, then close monitoring and consider consulting benign hematology team
Grade 2: Hold ICI; Trigger: if platelet count 50,000 to 75,000, then close monitoring and consult with benign hematology team
Grades 3-4: Discontinue ICI; Trigger: if platelet count < 50,000, then urgent consult with benign hematology team

Other rare hematological reactions:
Grades 1-4: ICI administration as per clinical judgement of treating physician and consider consult with benign hematology team

Neuromuscular/Joint/Cardiac:

All neuromuscular/joint/cardiac reactions:
Grade 1: ICI administration as per clinical judgement of treating physician and consider consulting rheumatology, neurology and/or cardiology teams
Grade 2: Hold ICI, consider starting immunosuppressive agents, and urgent consultation with rheumatology, neurology and/or cardiology teams
Grades 3-4: Hold ICI, hospitalization, start immunosuppressive agents, and emergent consultation with rheumatology, neurology and/or cardiology teams

Gastrointestinal:

Colitis:
Grade 1: ICI administration as per clinical judgement of treating physician; Trigger: if increase of < 4 stools per day over baseline, then close monitoring and consider consulting GI team
Grade 2: Hold ICI; Trigger: if increase of 4 to 6 stools per day over baseline, then start immunosuppressive agents, consult with GI team, and esophagogastroduodenoscopy (EGD)/colonoscopy

© 2021 Msaouel P et al. *JAMA Network Open.*

Grade 3: Hold ICI; Trigger: if increase of ≥ 7 stools per day over baseline, then hospitalization, start immunosuppressive agents, urgent consult with GI team, and EGD/colonoscopy
Grade 4: Discontinue ICI; Trigger: if life-threatening symptoms, then hospitalization, start immunosuppressive agents, emergent consult with GI team, and EGD/colonoscopy

Hepatitis:
Grade 1: Continue ICI; Trigger: if alanine transaminase (ALT) and/or aspartate transaminase (AST) between 1.5 to 3.0x baseline, then continue to monitor closely
Grade 2: Hold ICI; Trigger: if ALT and/or AST > 3.0 to 5.0x baseline, then start immunosuppressive agents and urgent consult with GI team
Grade 3-4: Discontinue ICI; Trigger: if ALT and/or AST > 5.0x baseline, then hospitalization, start immunosuppressive agents, and emergent consult with GI team

Skin:

Rash/inflammatory dermatitis:
Grade 1: Continue ICI; Trigger: if covers ≤ 10% body surface area (BSA), then close monitoring with topical treatment and consider consulting dermatology team.

Grade 2: Hold ICI; Trigger: if covers > 10% and ≤ 30% BSA, then urgent consult with dermatology team.
Grades 3-4: Discontinue ICI; Trigger: if covers > 30% BSA, then hospitalization, start immunosuppressive agents, and emergent consult with dermatology team.

Bullous dermatoses:
Grade 1: ICI administration per treating physician's clinical judgement. Trigger: if blisters cover < 10% BSA, then close monitoring and consult with dermatology team.
Grade 2: Hold ICI; Trigger: if blisters cover > 10% and < 30%, then start immunosuppressive agents and urgent consult with dermatology team.
Grades 3-4: Discontinue ICI; Trigger: if blisters cover > 30% BSA, then hospitalization, start immunosuppressive agents, and emergent consult with dermatology team.

SCARs: Stevens Johnson Syndrome (SJS), toxic epidermal necrolysis (TEN), acute generalized exanthematous pustulosis (AGEP), and drug reaction with eosinophilia and systemic symptoms (DRESS) (AKA: drug-induced hypersensitivity syndrome or DIHS)
Grade 1: N/A
Grade 2: N/A
Grades 3-4: Discontinue ICI; Trigger: if skin sloughing/mucous membrane detachment, hospitalization, start immunosuppressive agents and emergent consult with dermatology team.

Pulmonary:

© 2021 Msaouel P et al. *JAMA Network Open.*

Pneumonitis:
Grade 1: Hold ICI; Trigger: if asymptomatic or mildly symptomatic, then consult pulmonary team and work up to rule out alternate causes.
Grades 2-4: Discontinue ICI; Trigger: if symptomatic, then hospitalization, start immunosuppressive agents and urgent consult with pulmonary team.

Renal:

Nephritis:
Grade 1 (creatinine increased up to 1.5x of baseline): Continue ICI and repeat creatinine levels in 3 to 7 days; Trigger: if continued increase in creatinine, then order kidney biopsy even in setting of no urine white blood cells or red blood cells.
Grade 2 (creatinine increased 1.5x to 3x of baseline): Hold ICI, start immunosuppressive agents, and urgent consult with renal team, with kidney biopsy to be performed within 3 days of immunosuppressive agent initiation even in setting of bland urine.
Grade 3 (creatinine increased > 3x of baseline) and grade 4 (creatinine increased > 6x of baseline): Hold ICI and assess for dialysis indications (hyperkalemia, azotemia, acidosis, decreased urine output, volume overload); start immunosuppressive agents and urgent consult with renal team, with kidney biopsy to be performed within 3 days of immunosuppressive agent initiation even in setting of bland urine.

© 2021 Msaouel P et al. *JAMA Network Open.*

eAppendix 4. Representative quotes from telephone encounter notes.

Example communication leading to on-call physician intervention:

Spoke with patient, complaining of constant nausea/vomiting since starting therapy on Friday. The patient has been using the prescribed Zofran, but it is not helping. The patient has not eaten since Friday and is trying to keep liquids down but is really struggling to do that today. Denies fever or chills at this time. Advised patient we will page the on-call physician. Patient verbalized understanding of plan.

Example communication leading to earlier clinic visit:

Received message through the immunotherapy trial application from patient describing still having scrotal swelling. This was reported prior to this message. The patient's surgeon was notified and suggested the patient be seen sooner than currently scheduled. I have sent the team an email stating that the swelling is still present. I have also sent a message to the patient through the portal to watch for schedule changes, to notify me once they are completed, and to follow up with the triage nurse and the surgical team via the portal.

Example communications leading to reassuring advice:

Spoke to patient. The patient still reports nausea and loss of appetite. The patient is still not feeling well but was able to eat a little better. The patient had two episodes of loose stools. The patient's lab results were received today and emailed to the treating oncologist. The patient will be evaluated by their local oncologist. The patient states they will report any new symptoms.

Reviewed proper storage of research meds until research team can meet face to face to collect medication. Patient will e-mail me research med diary.

© 2021 Msaouel P et al. *JAMA Network Open.*

eAppendix 5. Formulas used to calculate the positive predictive value (PPV), negative predictive value (NPV), sensitivity, and specificity of the symptom alert thresholds.

$$PPV = \frac{\sum_N^1 \sum_J^1 \{\frac{1}{j} R^+{}_{nj} * I(AE\ with\ intervention\ within\ 2\ weeks)\}}{\sum_N^1 \sum_J^1 \frac{1}{j} R^+{}_{nj}}$$

$$NPV = \frac{\sum_N^1 \sum_K^1 \{\frac{1}{k} R^-{}_{nk} * I(no\ AE\ with\ intervention\ within\ 2\ weeks)\}}{\sum_N^1 \sum_K^1 \frac{1}{k} R^-{}_{nk}}$$

$$Sensitivity = \frac{\sum_N^1 \sum_L^1 \{\frac{1}{l} A^+{}_{nl} * I(symptom\ alert)\}}{\sum_N^1 \sum_L^1 \frac{1}{l} A^+{}_{nl}}$$

$$Specificity = \frac{\sum_N^1 \sum_M^1 \{\frac{1}{m} A^-{}_{nm} * I(no\ symptom\ alert)\}}{\sum_N^1 \sum_M^1 \frac{1}{m} A^-{}_{nm}}$$

Where:

AE: Adverse event of grade 2 or higher

$R^+{}_{nj}$***: patient n's jth report with an alert***

$R^-{}_{nk}$***: patient n's kth report without an alert***

$A^+{}_{nl}$***: patient n's lth report with linked*** $AE\ with\ intervention\ within\ 2\ weeks$

$A^-{}_{nm}$***: patient n's mth report without linked***

$AE\ with\ intervention\ within\ 2\ weeks$

© 2021 Msaouel P et al. *JAMA Network Open.*

eREFERENCES:

1. Dueck AC, Mendoza TR, Mitchell SA, et al. Validity and Reliability of the US National Cancer Institute's Patient-Reported Outcomes Version of the Common Terminology Criteria for Adverse Events (PRO-CTCAE). JAMA Oncol 2015;1:1051-9.
2. Gilbert A, Francischetto EO, Blazeby J, et al. Choice of a patient-reported outcome measure for patients with anal cancer for use in cancer clinical trials and routine clinical practice: a mixed methods approach. Lancet 2015;385 Suppl 1:S38.
3. Taarnhoj GA, Lindberg H, Dohn LH, et al. Electronic reporting of patient-reported outcomes in a fragile and comorbid population during cancer therapy - a feasibility study. Health Qual Life Outcomes 2020;18:225.
4. Tolstrup LK, Bastholt L, Zwisler AD, Dieperink KB, Pappot H. Selection of patient reported outcomes questions reflecting symptoms for patients with metastatic melanoma receiving immunotherapy. J Patient Rep Outcomes 2019;3:19.
5. Frazier L, Taft L, Roeper T, Clifton C, Ehrlich K. Parallel structure: a source of facilitation in sentence comprehension. Mem Cognit 1984;12:421-30.
6. National Cancer Institute. Common terminology criteria for adverse events : (CTCAE) version 5.0 (Accessed on March 09, 2018). 2017.

© 2021 Msaouel P et al. *JAMA Network Open.*
